# Deep learning predicts stent implantation in borderline coronary lesions from angiography

**DOI:** 10.1038/s41746-026-02779-z

**Published:** 2026-05-22

**Authors:** Jingsong Xia, Di Zhao, Yiming Zhang, Leilei Chen, Zhenhua Yang, Dengqing Shi, Chao Liu, Haoyu Meng, Liansheng Wang, Jiabao Liu

**Affiliations:** 1 Department of Cardiology, The First Affiliated Hospital with Nanjing Medical University, Nanjing, China; 2 The Second Clinical Medical College, Nanjing Medical University, Nanjing, China; 3 Department of Cardiology, The First Affiliated Hospital of Anhui Medical University, Hefei, China; 4 Ningxia Hui Autonomous Region Hospital of Traditional Chinese Medicine, Ningxia Hui Autonomous Region Academy of Traditional Chinese Medicine, Yinchuan, China

**Keywords:** Cardiology, Computational biology and bioinformatics, Diseases

## Abstract

Accurate evaluation of coronary intermediate lesions (50–70% stenosis) is essential for stent decision-making, yet conventional angiography remains subjective and adjunctive tests like FFR are often invasive or costly. In this retrospective multicenter study of 1298 patients, we developed an attention-enhanced deep learning model using Improved_EfficientNet with a Convolutional Block Attention Module to predict stent necessity directly from coronary angiography images. The model utilized multimodal labels from FFR, IVUS, and OCT as reference standards during training. In internal validation, the model achieved an accuracy of 0.976 and an F1-score of 0.971. External validation across independent institutions demonstrated robust performance with an accuracy of 0.807 and an AUC of 0.897. Grad-CAM visualization confirmed that the model focuses on clinically relevant stenotic regions, showing high alignment with expert interpretations. These results suggest that the proposed model can effectively integrate anatomical and functional information to provide real-time decision support, potentially reducing the need for invasive adjunctive testing and enhancing precision in interventional cardiology.

## Introduction

Coronary artery disease (CAD), characterized by atherosclerotic narrowing of the coronary arteries, remains a dominant challenge to global public health. According to the Global Burden of Disease (GBD) 2021 study, ischemic heart disease was the second leading cause of disability-adjusted life years (DALYs) globally in 2021, contributing 188.3 million DALYs (95% UI 176.7–198.3), surpassed only by COVID-19 during the pandemic peak^1^. In China, with population aging and lifestyle changes, both the incidence and mortality of CAD continue to rise^2–4^. Epidemiological studies indicate that the number of CAD patients in China has already exceeded 10 million and is projected to increase rapidly in the coming years^5^. Among patients requiring coronary revascularization, the decision of whether and when to implant a stent represents one of the most consequential clinical judgments in interventional cardiology, directly influencing patient outcomes, procedural risk, and long-term prognosis. In particular, for patients with coronary stenosis in the “borderline range” of 50–70% by Quantitative Coronary Angiography (QCA), accurate assessment of lesion characteristics and determination of the necessity for stent implantation are directly associated with prognosis and quality of life. Thus, evaluation and management of borderline lesions represent a major challenge in cardiovascular clinical practice^6^.

Coronary angiography (CAG), the clinical gold standard for CAD diagnosis and treatment decision-making, is widely used to assess the severity of coronary stenosis^7–9^. However, CAG has intrinsic limitations in the assessment of intermediate lesions, as visual estimation of stenosis severity is subject to inter-observer variability and projection artifacts^10^. To address specific diagnostic uncertainties that CAG alone cannot resolve, clinicians may employ adjunctive modalities with distinct clinical purposes: OCT and IVUS provide high-resolution assessment of plaque morphology, minimal lumen area (MLA), and vessel wall characteristics, including rupture, erosion, and thin-cap fibroatheroma; FFR quantifies the hemodynamic significance of a lesion by measuring the trans-stenotic pressure gradient, enabling physiologically guided revascularization decisions. These techniques provide complementary structural or functional insights into coronary lesions, thereby improving diagnostic objectivity^11–13^. Nevertheless, OCT and IVUS are invasive imaging methods associated with procedural risks and high costs, whereas FFR requires pharmacological induction and prolongs procedural time, often leading to reduced patient compliance^14^. Under the constraints of limited medical resources, an urgent clinical need exists for a strategy capable of accurately evaluating borderline lesions and predicting stent implantation based solely on CAG images^15,16^.

Given these diagnostic and resource constraints, a fundamental clinical gap exists: when borderline coronary lesions are identified on routine CAG, visual interpretation alone cannot reliably determine hemodynamic significance or guide revascularization decisions, yet adjunctive physiological or structural assessment is not universally available or feasible. This gap creates a category of patients whose optimal management remains uncertain at the time of catheterization. An AI model capable of extracting latent structural and functional diagnostic patterns directly from angiographic images could therefore provide clinically meaningful decision-support at precisely this juncture—before adjunctive testing is performed or when such testing is unavailable. In the present study, diagnostic conclusions derived from FFR, OCT, and IVUS were used as reference labels during model training, enabling the model to distill multimodal structural and functional insights into a streamlined angiographic inference engine. The trained model can thereby leverage routine CAG images to provide a surrogate for the diagnostic information typically obtained from multimodal assessments, supporting more informed stent implantation decisions without escalating procedural cost or complexity.

In recent years, the rapid advancement of artificial intelligence, particularly deep learning, has created new opportunities for intelligent interpretation of medical imaging^17^. Convolutional neural networks, as the cornerstone of deep learning, have achieved breakthroughs in image recognition and classification tasks, with increasing applications in radiology, pathology, and cardiovascular imaging. For instance, AI has demonstrated significant advantages in automated electrocardiogram analysis, coronary CT stenosis detection, and cardiac MRI functional assessment^18–23^. Meanwhile, the introduction of attention mechanisms has further enhanced the ability of deep learning models to capture critical image regions, enabling them not only to “see” but also to “focus” on the most relevant features^24,25^. Representative modules, such as squeeze-and-excitation (SE) attention and the convolutional block attention module (CBAM), have shown remarkable performance in medical image analysis^26–28^. In the domain of invasive coronary imaging, deep learning has also been applied to automated OCT plaque classification, IVUS-based MLA estimation, and angiography-derived fractional flow reserve computation, demonstrating the feasibility of AI-assisted functional lesion assessment across multiple coronary imaging modalities^15,16^. Despite the widespread clinical use of CAG, few studies have combined deep learning and attention mechanisms for intelligent decision support in borderline lesion assessment using multimodal imaging, leaving a significant research gap in this domain^29,30^.

**Fig. 1:**
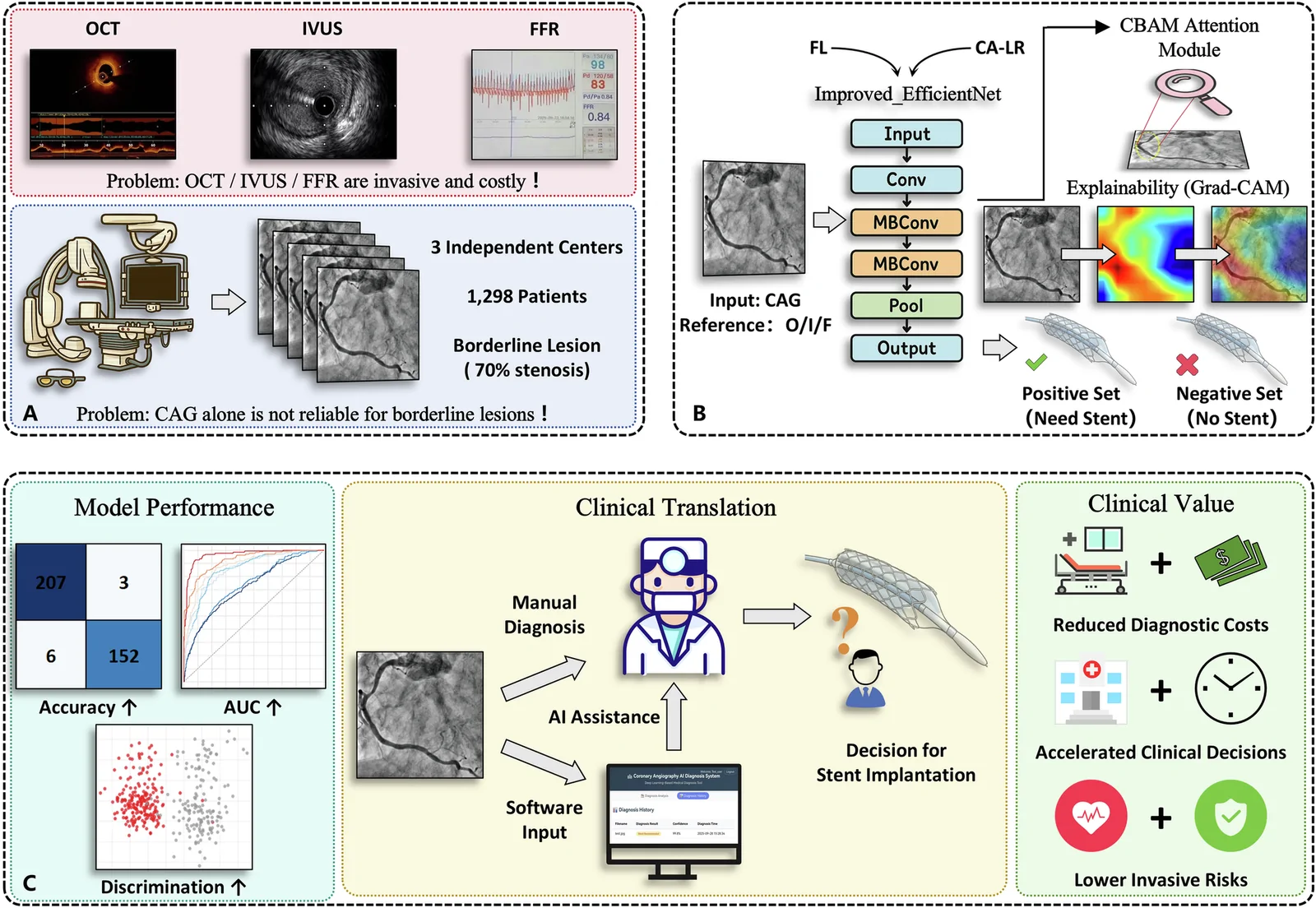
Flowchart of the study. **A** Problem description. This study included a total of 1298 patients from 3 independent centers. OCT, IVUS, and FFR are invasive and costly imaging modalities for assessing coronary lesions, while CAG alone is not reliable for borderline lesions with approximately 70% stenosis. **B** Model architecture. The Improved_EfficientNet backbone integrates CBAM attention module, CA-LR (channel attention with label refinement), and FL (focal loss) for enhanced feature extraction. Input is CAG images with references from OCT/IVUS/FFR; processing includes Conv, MBConv, and Pool layers; output classifies into Positive Set (Need Stent) or Negative Set (No Stent), with explainability via Grad-CAM heatmaps highlighting key lesion areas. **C** Model performance, clinical translation, and value. Performance metrics include confusion matrix, AUC curves, and discrimination scatter plots; clinical translation flows from manual diagnosis to AI assistance via software for stent implantation decisions; clinical value encompasses reduced diagnostic costs, accelerated decisions, and lower invasive risks.

This study proposes an attention-enhanced deep learning model tailored to the complex challenge of managing borderline coronary lesions by leveraging CAG imaging for intelligent prediction of stent implantation decisions (Fig. 1). It is important to note that OCT, IVUS, and FFR measurements were not used as model inputs at any stage. Instead, their diagnostic conclusions—together with guideline-concordant clinical treatment decisions when objective assessments were unavailable—served exclusively as reference labels for model training and supervision. This multimodal-label-guided paradigm enables the trained model to internalize diagnostic knowledge derived from multiple modalities while requiring a CAG image at the point of clinical inference, thereby preserving clinical feasibility without the additional costs and procedural risks associated with invasive adjunctive assessments. Specifically, this work integrates multimodal labels derived from OCT, IVUS, and FFR as reference standards, enabling cross-modal knowledge transfer that allows the model to learn both morphological and functional diagnostic features from CAG images, thereby improving the accuracy and robustness of borderline lesion prediction. Furthermore, CBAM was embedded into the feature extraction network to enhance the model’s sensitivity to hemodynamically significant regions and subtle boundary changes, ensuring precise identification of clinically relevant features. To improve interpretability and clinical trust, visualization techniques such as Grad-CAM were employed, confirming that the model’s attention regions highly overlapped with cardiologists’ diagnostic focus. Finally, the developed model demonstrates strong translational potential by enabling CAG-based prediction of stent implantation decisions, effectively simulating the diagnostic insights of FFR, OCT, and IVUS using a single frame from catheter-based x-ray CAG, thereby reducing dependence on invasive examinations, lowering clinical workload and variability, and offering real-time decision support for interventional cardiology.

## Results

### Baseline characteristics

A total of 1298 patients adhering to the predefined inclusion and exclusion criteria were enrolled. The diagnostic modalities for establishing the reference standard labels were distributed as follows: FFR in 756 patients (58.2%), IVUS in 312 patients (24.0%), and OCT in 230 patients (17.8%). This composition underscores our hierarchical labeling strategy, which prioritizes physiological assessment (FFR) over structural imaging. Following the integrated multi-modality adjudication, 669 patients (51.5%) were categorized into the “Need Stent” group (positive set), and 629 (48.5%) into the “No Stent” group (negative set), yielding a well-balanced cohort for deep learning.

The baseline demographic and clinical profiles are comprehensively summarized in Table 1. Comparative analysis revealed that key cardiovascular risk factors—including age (63.0 ± 10.9 vs. 62.5 ± 10.8 years, *p* = 0.6838), gender, hypertension, diabetes, and smoking status—were well-balanced between the two groups (all *p* > 0.05). Similarly, no significant difference was observed in baseline left ventricular ejection fraction (LVEF). Notably, while the groups were generally homogeneous, a significant difference was observed in clinical presentation (*p* = 0.0257); patients in the “Need Stent” group exhibited a higher prevalence of acute coronary syndromes (NSTEMI: 15.4% vs. 12.6%; STEMI: 7.0% vs. 4.9%) compared to those with stable coronary artery disease. All continuous variables were compared using Student’s t-test or Mann-Whitney U test, while categorical variables were assessed via the *χ*² test.

**Table 1:** Baseline clinical and angiographic characteristics of patients in the positive and negative sets

	Positive set (*n* = 669)	Negative set (*n* = 629)	*P*-value
Age (mean ± SD, years)	**63.0** **±** **10.9**	**62.5** **±** **10.8**	0.6838
<60 years	237 (35.5)	225 (35.7)	
≥60 years	432 (64.5)	404 (64.3)	
Gender			0.5779
Male	506 (75.7)	459 (72.9)	
Female	163 (24.3)	170 (27.1)	
Hypertension			0.1346
Yes	415 (62.1)	443 (70.5)	
No	254 (37.9)	186 (29.5)	
Diabetes			0.9527
Yes	194 (29.0)	181 (28.7)	
No	475 (71.0)	448 (71.3)	
Smoking			0.2499
Yes	320 (47.9)	259 (41.1)	
No	349 (52.1)	370 (58.9)	
Drinking			0.3506
Yes	229 (34.3)	248 (39.5)	
No	440 (65.7)	381 (60.5)	
LVEF (mean ± SD, %)	**53.2** ± **18.5**	**54.8** ± **16.4**	0.6604
≤50%	223 (33.3)	204 (32.4)	
>50%	446 (66.7)	425 (67.6)	
Clinical presentation			0.0257
Stable CAD	362 (54.1)	391 (62.2)	
Unstable Angina	157 (23.5)	128 (20.3)	
NSTEMI	103 (15.4)	79 (12.6)	
STEMI	47 (7.0)	31 (4.9)	

### Clinical evaluation of the model

For the clinical evaluation of stent implantation decision-making in coronary intermediate lesions, we assessed the proposed Improved_EfficientNet model under three validation paradigms: cross-validation (CV), internal validation (IV), and external validation (EV). These evaluations were benchmarked against several established architectures, including Swin-T, InceptionV3, DenseNet121, ResNet50, VGG11, EfficientNet, and MobileNetV2, to demonstrate the superior diagnostic accuracy and robustness of our approach.

Quantitative performance metrics across the three validation paradigms are summarized in Table 2, including accuracy, recall, F1-score, positive predictive value (PPV), and negative predictive value (NPV), each reported with its corresponding 95% confidence interval (CI). The proposed Improved_EfficientNet consistently outperformed all baseline architectures across every metric and validation setting. In the CV and IV phases, the model achieved exceptional stability, with accuracies of 0.976 (95% CI: 0.955–0.993) and 0.976 (95% CI: 0.959–0.989), respectively. Crucially, in the challenging EV setting, Improved_EfficientNet maintained robust diagnostic performance with an accuracy of 0.807 (95% CI: 0.745–0.863) and an F1-score of 0.789 (95% CI: 0.712–0.857). The clinical reliability of the model is further underscored by its superior predictive values. In the EV cohort, Improved_EfficientNet yielded a PPV of 0.866 and an NPV of 0.766, significantly surpassing the next best performing model, Swin-T (PPV: 0.646; NPV: 0.722). Notably, the 95% CIs for accuracy and F1-score of the Improved_EfficientNet in the CV and IV settings did not overlap with those of any comparator models, demonstrating a statistically significant improvement in identifying lesions requiring stent implantation. Even under the rigorous EV paradigm, the model showed a 13.0% improvement in F1-score over Swin-T (0.789 vs. 0.698), reinforcing its generalizability and potential for standardized clinical decision support across different medical centers.

**Table 2:** Comparative performance of deep learning models across cross-validation, internal validation, and external validation

Model	Test	Accuracy (95% CI)	Recall (95% CI)	F1-Score (95% CI)	PPV (95% CI)	NPV (95% CI)
Improved_EfficientNet	CV	0.976 (0.955–0.993)	0.965 (0.931–0.993)	0.975 (0.956–0.993)	0.986 (0.964–0.996)	0.966 (0.922–0.985)
	IV	0.976 (0.959–0.989)	0.962 (0.931–0.988)	0.971 (0.951–0.988)	0.981 (0.956–0.989)	0.972 (0.940–0.987)
	EV	0.807 (0.745–0.863)	0.726 (0.629–0.820)	0.789 (0.712–0.857)	0.866 (0.779–0.940)	0.766 (0.671–0.840)
Swin-T	CV	0.847 (0.806–0.889)	0.923 (0.877–0.963)	0.858 (0.816–0.895)	0.801 (0.740–0.861)	0.910 (0.846–0.949)
	IV	0.889 (0.856–0.918)	0.949 (0.911–0.980)	0.879 (0.843–0.912)	0.820 (0.765–0.872)	0.957 (0.917–0.978)
	EV	0.677 (0.602–0.745)	0.763 (0.662–0.854)	0.698 (0.616–0.773)	0.646 (0.544–0.739)	0.722 (0.605–0.815)
InceptionV3	CV	0.774 (0.726–0.823)	0.672 (0.595–0.750)	0.748 (0.688–0.804)	0.844 (0.780–0.904)	0.728 (0.657–0.789)
	IV	0.853 (0.815–0.889)	0.825 (0.761–0.883)	0.828 (0.781–0.871)	0.833 (0.773–0.891)	0.869 (0.817–0.908)
	EV	0.652 (0.578–0.727)	0.527 (0.417–0.635)	0.601 (0.504–0.694)	0.704 (0.581–0.813)	0.619 (0.522–0.708)
ResNet50	CV	0.736 (0.684–0.785)	0.603 (0.523–0.684)	0.694 (0.626–0.760)	0.819 (0.747–0.888)	0.689 (0.619–0.752)
	IV	0.774 (0.731–0.815)	0.741 (0.671–0.804)	0.738 (0.681–0.794)	0.736 (0.667–0.806)	0.804 (0.745–0.852)
	EV	0.696 (0.621–0.764)	0.593 (0.486–0.703)	0.661 (0.567–0.750)	0.750 (0.641–0.847)	0.660 (0.561–0.746)
DenseNet121	CV	0.726 (0.674–0.778)	0.548 (0.468–0.629)	0.665 (0.595–0.732)	0.850 (0.773–0.921)	0.666 (0.598–0.729)
	IV	0.755 (0.712–0.799)	0.494 (0.416–0.567)	0.633 (0.563–0.699)	0.886 (0.818–0.946)	0.714 (0.659–0.764)
	EV	0.627 (0.553–0.702)	0.377 (0.277–0.479)	0.500 (0.393–0.603)	0.753 (0.614–0.872)	0.587 (0.498–0.671)
VGG11	CV	0.615 (0.559–0.670)	0.272 (0.204–0.346)	0.413 (0.326–0.503)	0.866 (0.761–0.957)	0.568 (0.505–0.629)
	IV	0.668 (0.620–0.717)	0.261 (0.196–0.333)	0.402 (0.318–0.490)	0.892 (0.791–0.976)	0.637 (0.583–0.687)
	EV	0.615 (0.540–0.689)	0.288 (0.192–0.387)	0.423 (0.304–0.537)	0.819 (0.667–0.957)	0.571 (0.486–0.652)
EfficientNet	CV	0.493 (0.434–0.552)	0.549 (0.469–0.628)	0.520 (0.452–0.585)	0.495 (0.423–0.568)	0.492 (0.407–0.578)
	IV	0.655 (0.606–0.704)	0.691 (0.615–0.764)	0.633 (0.572–0.692)	0.585 (0.509–0.657)	0.727 (0.658–0.787)
	EV	0.602 (0.528–0.677)	0.611 (0.507–0.723)	0.603 (0.510–0.687)	0.597 (0.483–0.698)	0.607 (0.497–0.708)
MobileNetV2	CV	0.646 (0.590–0.701)	0.335 (0.264–0.418)	0.485 (0.402–0.575)	0.888 (0.800–0.964)	0.590 (0.526–0.651)
	IV	0.674 (0.625–0.720)	0.412 (0.336–0.494)	0.519 (0.439–0.597)	0.706 (0.613–0.793)	0.663 (0.605–0.716)
	EV	0.578 (0.503–0.652)	0.313 (0.211–0.420)	0.422 (0.302–0.537)	0.655 (0.500–0.794)	0.553 (0.465–0.638)

*CV* cross validation, *IV* internal validation, *EV* external validation.

**Fig. 2:**
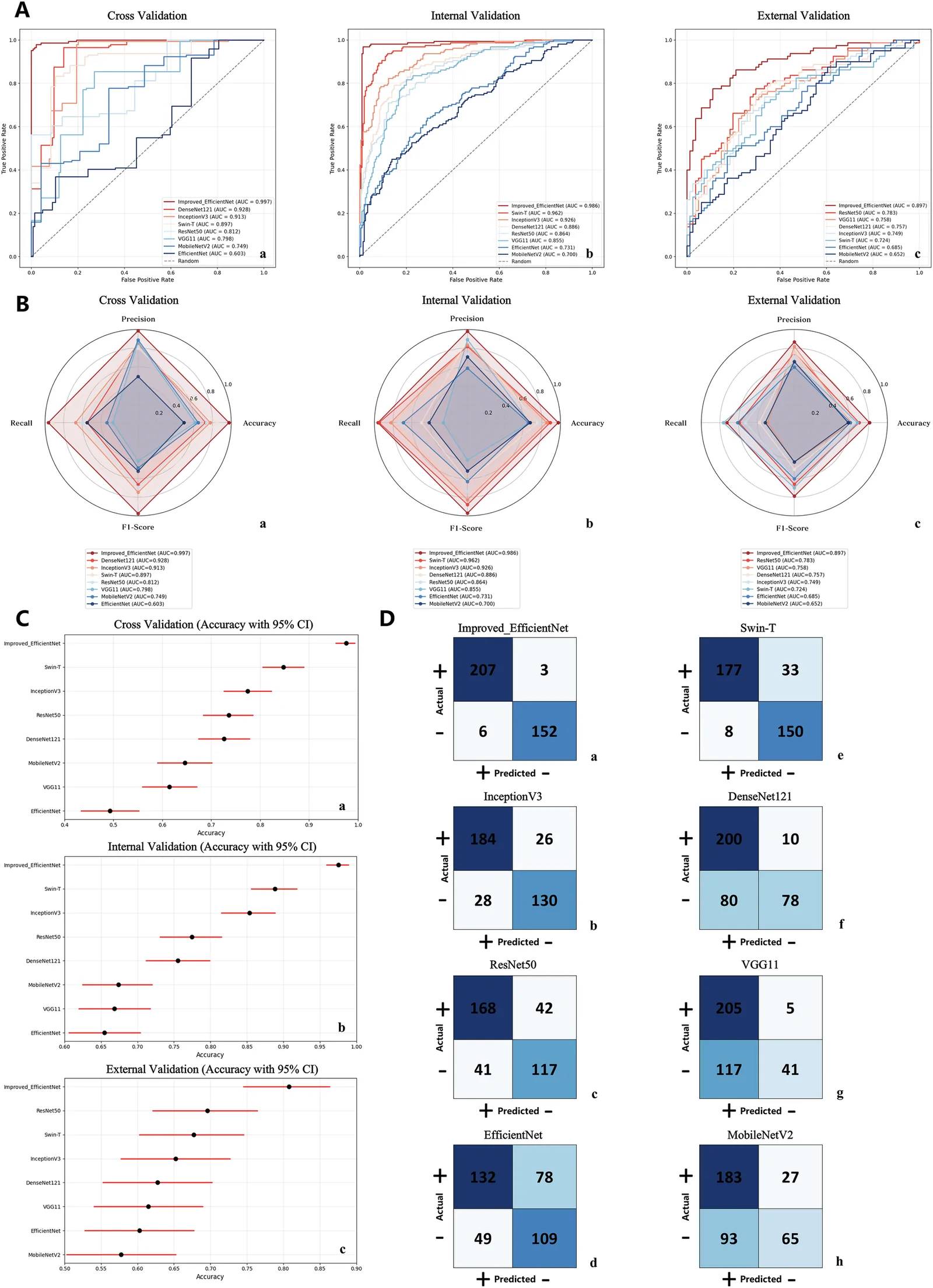
Model evaluation across validation cohorts. **A** ROC curves for Improved_EfficientNet and comparator models across cross-validation, internal validation, and external validation. The proposed model consistently achieved the highest AUC. **B** Radar plots of classification metrics (precision, recall, F1-score, and accuracy) in the three validation settings, highlighting balanced performance of Improved_EfficientNet. **C** Accuracy with 95% confidence intervals for all models in cross validation and internal validation cohorts, showing superior robustness of Improved_EfficientNet. **D** Confusion matrices of representative models in internal validation, demonstrating improved discrimination between Positive Set (Need Stent) and Negative Set (No Stent) with the proposed approach.

Figure 2A illustrates the ROC curves for all models under the three validation paradigms. Improved_EfficientNet consistently achieved the highest AUC values—0.997 in CV, 0.986 in IV, and 0.897 in EV—outperforming all benchmark models such as Swin-T (AUC: 0.897 in CV, 0.962 in IV, 0.724 in EV) and InceptionV3 (AUC: 0.913 in CV, 0.926 in IV, 0.749 in EV). Figure 2B qualitatively presents Accuracy, Precision, Recall, and F1-Score across all models, where Improved_EfficientNet demonstrated superior performance across CV, IV, and EV compared with other benchmarks. Figure 2C displays accuracy metrics with 95% CIs across CV, IV, and EV, sorted in descending order. Improved_EfficientNet achieved the highest accuracy among all comparator models in all three validation settings, with non-overlapping CIs indicating statistically significant superiority. Figure 2D shows confusion matrices for all models, providing fine-grained insights into classification errors. For Improved_EfficientNet (Fig. 2Da), the confusion matrix revealed the fewest off-diagonal misclassifications, with true positives dominating, in contrast to models such as ResNet 50 (Fig. 2Dc) and MobileNetV2 (Fig. 2Dh) that exhibited higher false negatives. This reflects the model’s effectiveness in reducing diagnostic errors in real-world clinical workflows, particularly for predicting stent implantation decisions in intermediate coronary lesions.

### Subgroup analysis by reference modality

**Fig. 3:**
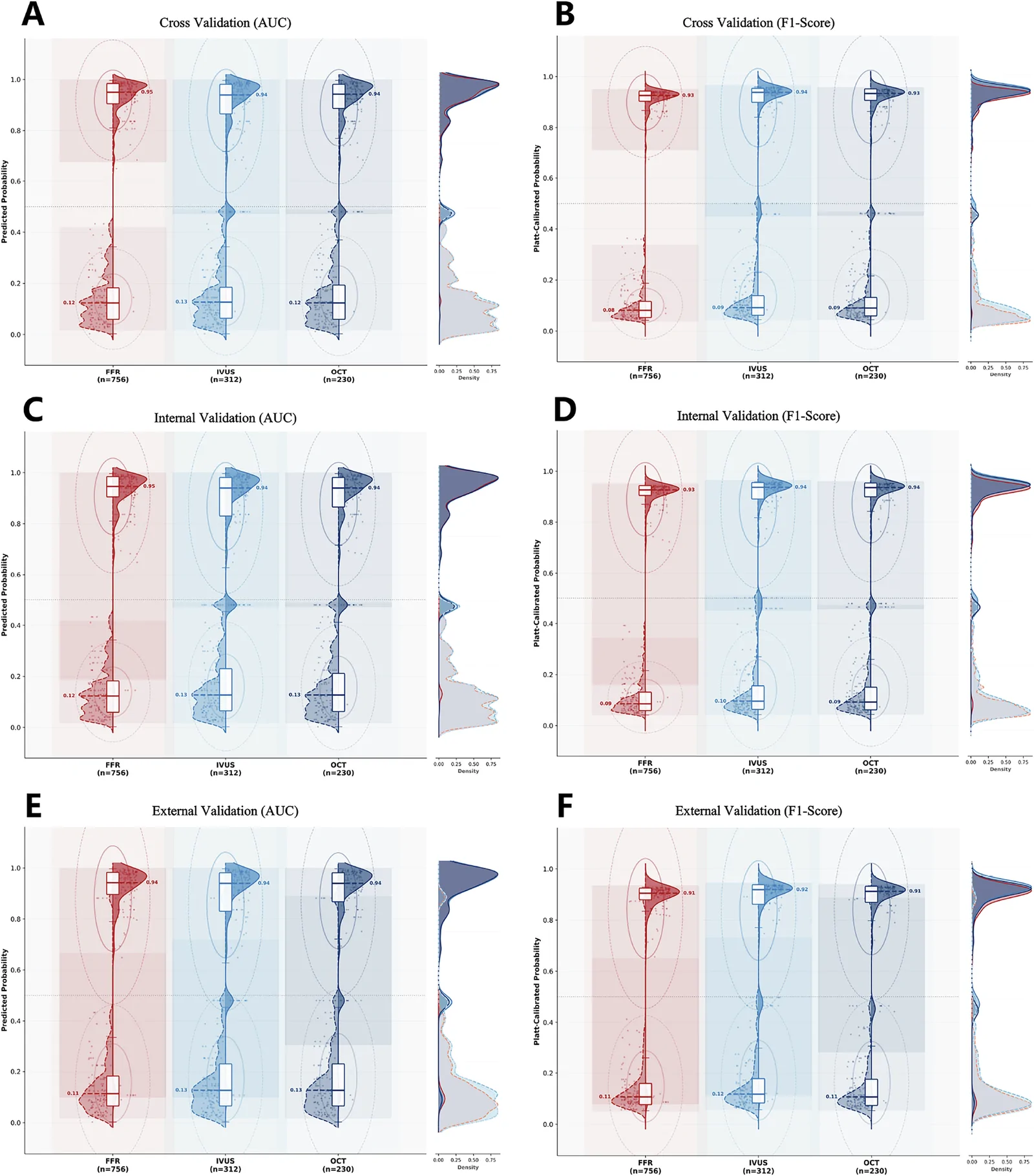
Reference-modality subgroup analysis of prediction probability distributions. **A**, **C**, **E** Subgroup probability distribution analysis under CV, IV, and EV based on AUC-oriented outputs. Cases were stratified by reference modality (FFR, *n* = 756; IVUS, *n* = 312; OCT, *n* = 230). Each panel presents mirrored KDE violin plots (bandwidth = 0.12) with embedded IQR box-and-whisker structures, jittered scatter points (≤150/class), 95% CI shaded bands, and ±1/2 SD concentric ellipses. Annotated values indicate the median predicted probability for each class. A right marginal density panel summarizes the overall score distribution across all subgroups. **B**, **D**, **F** Corresponding F1-oriented analysis under identical validation settings, with predicted probabilities Platt-calibrated prior to visualization (*y*-axis: Platt-Calibrated Probability). Graphical components are identical to A/C/E. Consistent distributional patterns across all three reference-modality subgroups and validation settings indicate stable model generalization, with no subgroup-specific performance collapse observed in the smaller IVUS or OCT strata.

To address the potential concern that model performance may be primarily driven by the predominant FFR-labeled subgroup rather than reflecting genuine multimodal learning, we conducted a stratified subgroup analysis examining prediction probability distributions separately across the three reference-modality groups (FFR, *n* = 756; IVUS, *n* = 312; OCT, *n* = 230). Figure 3A–F illustrates the predicted probability distributions for positive and negative samples within each subgroup under cross-validation, internal validation, and external validation settings, presented from both AUC-oriented and F1-score–oriented perspectives.

Across all three reference-modality subgroups, the model exhibited broadly consistent distributional patterns: positive samples clustered at high predicted probabilities and negative samples at low predicted probabilities, with clear separation between classes regardless of the reference modality used for labeling. Notably, despite the substantially smaller sample sizes of the IVUS (*n* = 312) and OCT (*n* = 230) subgroups relative to FFR (*n* = 756), no subgroup-specific distributional collapse or degradation in class separation was observed. Kolmogorov–Smirnov tests confirmed statistically significant separation between positive and negative probability distributions across all three subgroups in all validation settings (all *p* < 0.001). These findings indicate that the model generalizes effectively across different reference-modality subgroups rather than overfitting to the FFR-dominant labeling pattern, providing empirical evidence that the hierarchical multimodal labeling framework supports genuine cross-modal knowledge transfer rather than single-modality decision replication.

### Interpretability of the prediction process

To enhance clinical acceptance and transparency of the model, Grad-CAM was employed to visualize the decision-making process, complemented by feature-space dimensionality reduction for qualitative and quantitative evaluation. These approaches help to reveal the imaging regions the model attends to and assess their consistency with clinical interpretation.

**Fig. 4:**
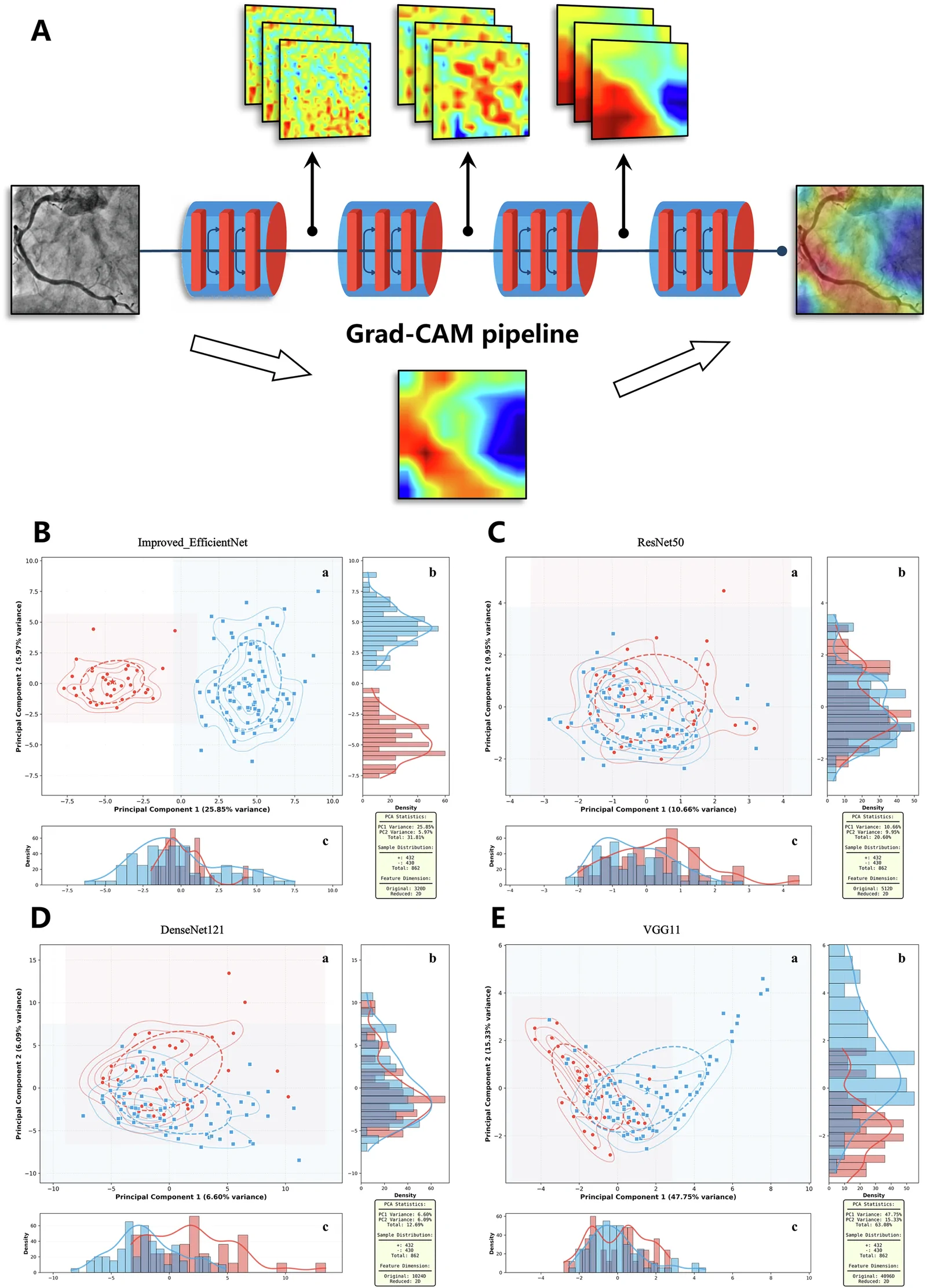
Visualization of model workflow and feature representation. **A** Grad-CAM interpretability pipeline of the proposed Improved_EfficientNet model. The original coronary angiography image is processed through successive convolutional feature extraction stages. Intermediate feature maps generated at different network depths illustrate the hierarchical abstraction of vascular patterns. The final Grad-CAM heatmap highlights lesion-relevant regions contributing most strongly to the classification decision, demonstrating the spatial interpretability of the model. **B**–**E** Two-dimensional PCA visualization of latent feature representations extracted from four convolutional neural network architectures (Improved_EfficientNet, ResNet50, DenseNet121, and VGG11). Each panel contains three complementary components: a) PCA scatter plot of feature embeddings. Each point represents an individual angiographic sample projected into the two-dimensional PCA space. Red circles indicate Positive Set (Need Stent) cases, while blue squares indicate Negative Set (No Stent) cases. Solid contour lines represent kernel density estimation (KDE) of class distributions, illustrating the spatial concentration of feature clusters. Dashed ellipses indicate 95% confidence regions for each class, summarizing the covariance structure of the feature distribution. Star markers denote the centroid of each class cluster, representing the mean location of feature embeddings. b) Marginal density distribution along Principal Component 1 (PC1). Horizontal histograms and smoothed density curves illustrate the distribution of feature values along PC1 for each class, enabling visual comparison of class separation along the primary variance direction. c) Marginal density distribution along Principal Component 2 (PC2). Vertical histograms and density curves represent the distribution of features along PC2, revealing complementary class separability along the secondary variance direction. Compared with other architectures, Improved_EfficientNet demonstrates more compact and well-separated class clusters with reduced overlap between positive and negative samples, suggesting superior feature discriminability.

Figure 4A depicts the stepwise Grad-CAM process in detail. Starting from the raw CAG image on the left, the model extracts multiple intermediate feature maps. Corresponding heatmaps are stacked above, evolving from dispersed low-activation regions (predominantly blue, indicating low attention) to concentrated high-activation regions (yellow-to-red gradient, indicating high attention). The final output includes both the complete heatmap and its overlay on the CAG, precisely covering stenotic segments and adjacent regions, thereby highlighting the model’s focus on clinically relevant lesion sites such as the narrowest lumen and disturbed flow regions. This corresponds closely with cardiologists’ observations in OCT/IVUS examinations, suggesting that Improved_EfficientNet effectively simulates human visual interpretation logic.

In qualitative analysis, heatmaps for true-positive samples were primarily concentrated on the lesion core, with precise coverage and strong activation. In contrast, false positives or false negatives showed activations that diffused into artifacts, indicating potential areas for improvement through data augmentation or attention mechanism optimization. For quantitative assessment of clinical consistency, two independent cardiovascular experts performed blinded scoring on 100 Grad-CAM overlays (scoring scale: 0 = no consistency; 1 = partial consistency; 2 = high consistency). The experts’ average score was 1.734 ± 0.216, confirming strong alignment between the model’s attention regions and expert interpretation. This result enhances model interpretability and supports its utility in clinical decision-making.

To further investigate the distribution of features learned by the model, intermediate feature vectors (outputs of global average pooling) were extracted from the test set and subjected to dimensionality reduction using PCA. Figure 4B–E illustrates the two-dimensional PCA scatter plots of feature representations extracted by the Improved_EfficientNet and several baseline models, including ResNet50, DenseNet121, and VGG11. Red points denote positive samples (Need Stent), whereas blue points represent negative samples (No Stent). The baseline models exhibited limited class separability: both ResNet50 and DenseNet121 showed substantial overlap between positive and negative samples with indistinct decision boundaries, indicating weaker discriminative capability (Fig. 4Ca, b and Fig. 4Da, b). VGG11 and related architectures demonstrated a more linear distribution pattern, but the boundary between classes remained insufficiently defined, resulting in suboptimal separation (Fig. 4Ea-b.). In contrast, the Improved_EfficientNet achieved markedly superior class discrimination, with positive and negative samples forming compact and well-separated clusters, clearer class boundaries, and substantially enhanced overall separability compared with the baseline models (Fig. 4B).

### Performance and representation analysis of different multimodal models

**Fig. 5:**
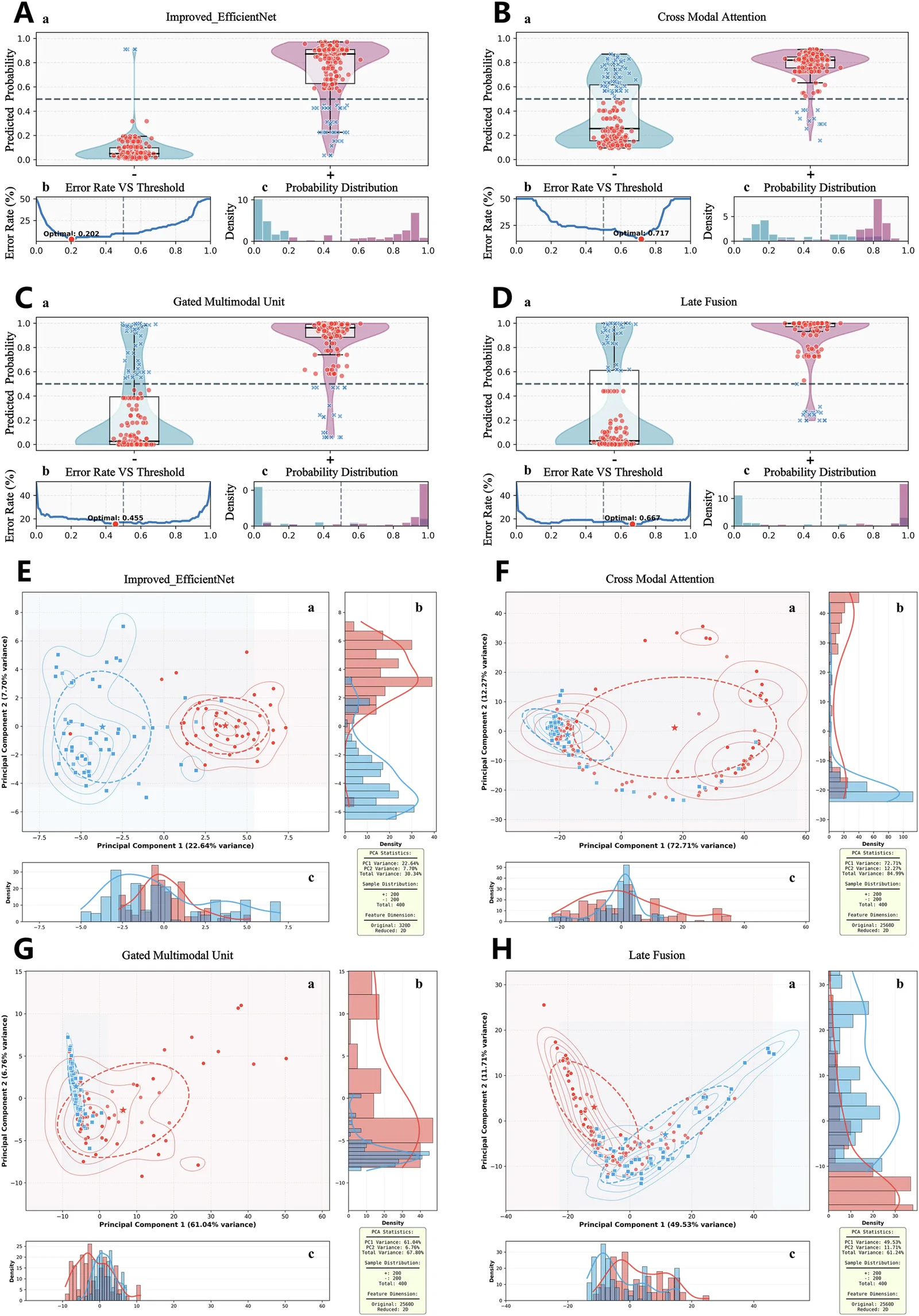
Decision boundary quality and multimodal fusion effectiveness. **A**–**D** Comparative evaluation of four multimodal fusion strategies: Improved_EfficientNet, Cross-Modal Attention, Gated Multimodal Unit (GMU), and Late Fusion. Each panel includes three components describing the classification behavior of the model: (a) Predicted probability distribution plot. Each point represents an individual sample’s predicted probability of belonging to the Positive Set (Need Stent). Red dots indicate correctly classified samples, whereas blue crosses indicate misclassified samples. Violin plots illustrate the probability distribution of predictions for positive and negative classes. Embedded box plots show the median and interquartile range of predicted probabilities. The horizontal dashed line represents the optimal classification threshold determined by minimizing the error rate. (b) Error rate versus threshold curve. The curve depicts how the overall classification error rate varies across different probability thresholds. The red marker identifies the optimal operating threshold, corresponding to the minimal classification error. (c) Probability density distribution. Histograms and smoothed density curves show the overall distribution of predicted probabilities for both classes. Better models exhibit clearer bimodal separation between positive and negative predictions. Among the evaluated strategies, Improved_EfficientNet achieves the lowest minimal error rate (optimal threshold = 0.202) and demonstrates improved separation between positive and negative prediction distributions. **E**–**H** Two-dimensional PCA projections of latent feature embeddings generated by the four multimodal fusion models. (a) PCA scatter plots display the spatial distribution of samples in the feature space. Red points denote Positive Set (Need Stent) samples, whereas blue points denote Negative Set (No Stent) samples. Solid density contours represent kernel density estimation of class distributions, while dashed ellipses indicate 95% confidence regions summarizing feature dispersion. Star markers denote the class centroids, representing the average feature location for each class. (b) Marginal histogram and density curve along PC1 illustrate the distribution of feature values along the primary principal component. (c) Marginal histogram and density curve along PC2 illustrate the distribution of feature values along the secondary principal component. The Improved_EfficientNet-based fusion model produces more compact feature clusters and clearer class separation, whereas alternative fusion strategies show increased overlap and less distinct decision boundaries.

Figure 5 summarizes the comparative performance of four multimodal fusion architectures integrating CAG images with clinical information. As shown in Fig. 5A–D, the proposed architecture achieved improved separation of predicted probabilities between negative and positive cases, with reduced overlap around the decision threshold (0.5). Correct predictions were tightly clustered near probability extremes, indicating high decision confidence. In contrast, Cross Modal Attention, GMU, and Late Fusion exhibited broader probability distributions with substantial overlap in the intermediate range (0.2–0.8), reflecting increased prediction uncertainty.

Threshold sensitivity analysis further supported these observations. As illustrated by the Error Rate–Threshold curves (Fig. 5A–Db), the optimal decision thresholds varied markedly across multimodal architectures. The optimal threshold of the Improved_EfficientNet was lower than those of Cross Modal Attention, GMU, and Late Fusion, while still achieving a comparatively low minimal error rate. Moreover, the proposed model demonstrated a relatively flat error curve around its optimal operating point, indicating stable discriminative performance across a wide range of thresholds and reduced sensitivity to threshold selection. By contrast, the alternative fusion strategies achieved minimal error rates comparable to that of the Improved_EfficientNet, their error curves were steeper near the optimal thresholds and their optimal decision thresholds were higher than that of the Improved_EfficientNet, suggesting heightened dependence on precise threshold tuning. Such threshold instability may limit model generalizability and reliability when deployed under fixed decision thresholds in the real-world clinical settings.

Probability density histograms further revealed pronounced differences in prediction confidence distributions among models (Fig. 5A–Dc). In the Improved_EfficientNet, negative samples were predominantly concentrated in the low-probability range (0–0.2), whereas positive samples spanned a broader range and formed a distinct peak in the high-probability interval (0.8–1.0), indicating effective class separation. In contrast, other multimodal models exhibited varying degrees of overlap between positive and negative samples in the intermediate probability range, along with pronounced peaks near both boundary values (0 and 1), suggesting the coexistence of overconfidence and localized misclassification.

Figure 5E–H presents PCA-based visualizations of feature representations learned by different models. The proposed architecture produced compact and well-separated class clusters with minimal overlap, particularly along the second principal component (Fig. 5Eb). Confidence ellipses and marginal density plots further confirmed superior class separability and reduced intra-class variance (Fig. 5Ea). In contrast, other fusion strategies yielded more dispersed feature distributions with substantial inter-class mixing, especially for GMU (Fig. 5Ga, b). Collectively, these findings indicate that multimodal performance is strongly dependent on fusion architecture rather than modality inclusion alone. Suboptimal integration of clinical information may attenuate the discriminative power of imaging features, underscoring the necessity of carefully designed fusion strategies to achieve robust and reliable decision support in coronary artery disease assessment.

### Software implementation and interface demonstration

Given the superior performance of the proposed model, we translated it into clinical practice by encapsulating the trained best-performing model into a clinical auxiliary software, the CAG AI Diagnosis System.

To evaluate the feasibility of real-time clinical deployment, we rigorously benchmarked the model’s computational efficiency. On a standard clinical workstation equipped with a mid-range GPU (NVIDIA RTX 3060 Ti), the total end-to-end inference time per case was 1.1 ± 0.2 s (range: 0.9–2.1 s). This comprehensive metric encompasses the entire automated pipeline: DICOM metadata parsing, standardized preprocessing (resizing and CLAHE), the model’s forward pass, and the simultaneous generation of interpretable Grad-CAM heatmaps. Notably, the system maintained a high-efficiency profile even in CPU-only mode (Intel i7-12700K), yielding an inference time of 3.8 ± 0.6 s, which remains well within the acceptable window for point-of-care diagnostic support.

**Fig. 6:**
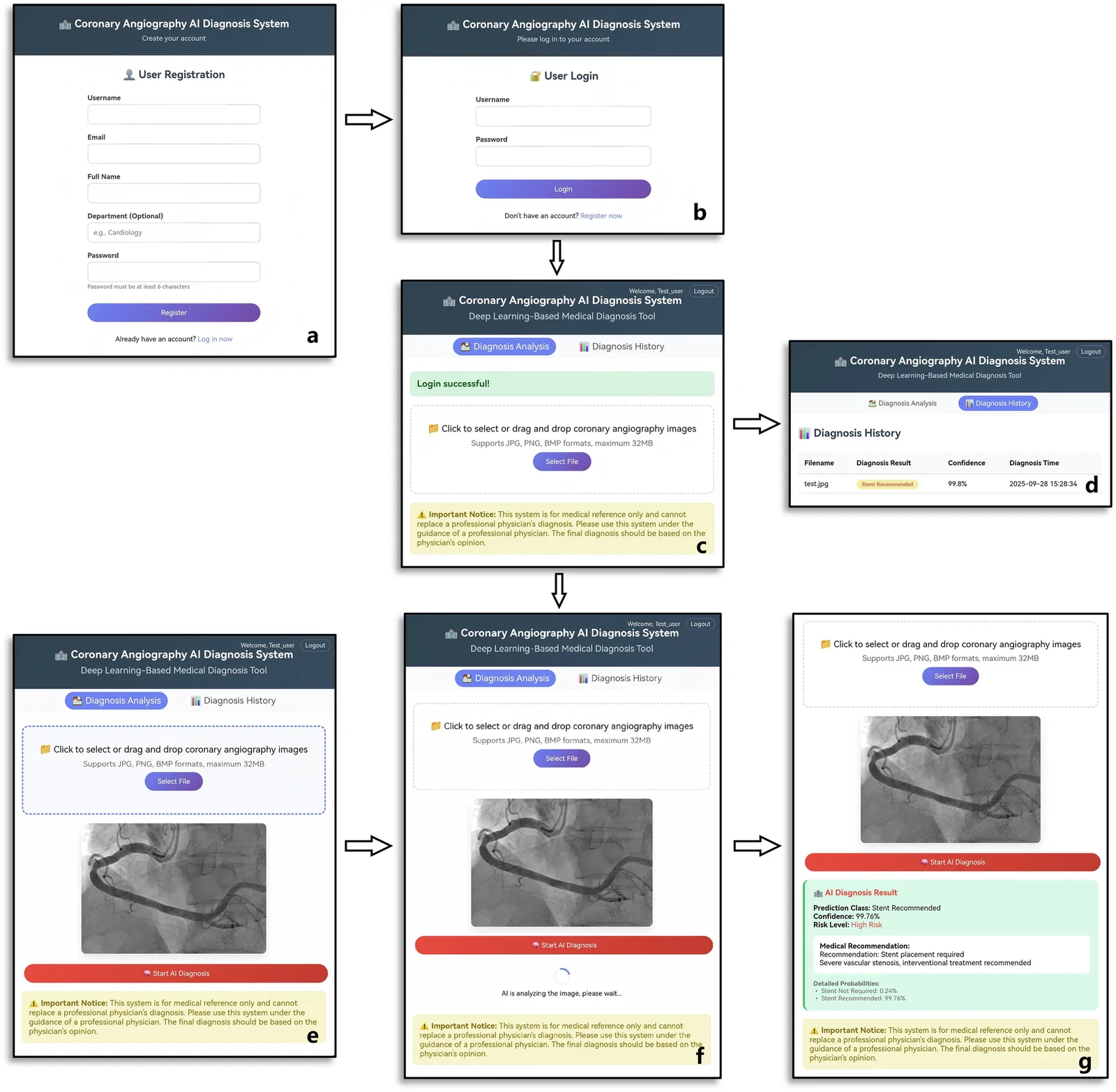
Workflow of the coronary angiography AI diagnostic system. **a** User registration interface, allowing clinicians to create an account by providing essential information. **b** User login page for secure access to the system. **c** Main dashboard after successful login, providing two key modules: Diagnosis Analysis for uploading and analyzing coronary angiography (CAG) images, and Diagnosis History for reviewing past results. **d** Diagnosis history interface, displaying previous cases with diagnosis result, confidence score, and timestamp. **e** Image upload panel, where CAG images are selected or dragged and dropped for analysis. **f** Diagnostic process interface, showing the uploaded angiography image and activation of the AI-based analysis pipeline. **g** Final diagnostic report generated by the AI system, including prediction result (Need Stent vs. No Stent), confidence level, and medical recommendations.

These technical parameters were encapsulated into this web-based, cross-platform application, which is compatible with macOS, Windows, as well as iOS and Android platforms. It is designed to provide intelligent diagnostic support for coronary intermediate lesions by integrating core functionalities such as user management, image uploading, AI diagnostic analysis, and historical record retrieval. The user interface adopts an intuitive flow-oriented design, guiding users from registration to diagnostic result output in a closed-loop process (Fig. 6). By delivering near-instantaneous diagnostic insights—bridging the gap between raw image acquisition and functional interpretation—the system demonstrates significant potential for reducing procedural delays and assisting in-room decision-making during coronary interventions. The functional workflow is as follows:

User Registration (Fig. 6a): New users create an account by entering a username, email, full name, department, and password. Upon registration, they are redirected to the login interface.

User Login (Fig. 6b): Existing users log in with their credentials. Upon successful authentication, they enter the main interface, which displays two core modules: Diagnosis Analysis and Diagnosis History.

Image Upload (Fig. 6c): Users click “Click to select or drag and drop coronary angiography images” to upload CAG images in JPG, PNG, or BMP formats, with a maximum size of 32 MB. A preview function is provided, alongside an important disclaimer: the system is for medical reference only and cannot replace professional physician judgment.

Diagnosis History (Fig. 6d): Users can review previous diagnostic records, including filename, diagnostic results, confidence score, and timestamp, facilitating case tracking and retrospective review.

Initiating Diagnosis (Fig. 6e): After uploading images, users click Start AI Diagnosis to initiate AI analysis. The system processes the image automatically, transitioning to the analysis progress interface.

Analysis Progress (Fig. 6f): A progress bar indicates “AI is analyzing the image…,” while reiterating that the system serves only as an auxiliary tool and cannot replace physician judgment.

Diagnostic Output (Fig. 6g): Upon completion, a detailed report is generated, including AI diagnostic results (e.g., Prediction Class: Stent Recommended), risk stratification (e.g., Risk Level: High), medical recommendations (e.g., Medical Recommendation: Severe vascular stenosis, interventional treatment recommended), and probability distribution (e.g., stent implantation probability of 99.75%). Results are color-coded (e.g., red for high risk, green for recommended treatment) and explicitly emphasize the primacy of physician judgment.

The software prioritizes security and compliance, requiring user authentication for all operations and repeatedly reminding users of the advisory nature of AI outputs. Through this design, the system can be seamlessly integrated into clinical workflows, assisting physicians in the rapid assessment of intermediate coronary lesions, reducing subjective bias, and improving decision-making efficiency. The software has obtained copyright registration from the National Copyright Administration of China (Registration No.2025SR2285239), ensuring intellectual property protection and regulatory compliance. Future directions include expansion to more mobile platforms and integration with hospital information systems.

## Discussion

In this study, we propose for the first time—and systematically validate—a deep learning model incorporating an attention mechanism, which is capable of intelligently predicting, from CAG images alone, whether patients with borderline lesions require stent implantation. Compared with conventional decision-making that relies on operators’ subjective experience, our model demonstrates significantly superior discriminative performance in both internal and external validation cohorts: metrics such as AUC, accuracy, and recall all exceed conventional operator assessment levels, thereby convincingly establishing the algorithm’s robustness and reliability. More importantly, via Grad-CAM visualization, we reveal that the model focuses its decision-making on critical regions concentrated around the stenotic segment and adjacent zones of hemodynamic perturbation. This “interpretable pattern” is highly consistent with the logical reasoning used by interventional cardiology experts in functional assessment, further confirming the model’s clinical rationality and generalizability.

The innovation of this work lies in that, without requiring additional invasive examinations such as OCT, IVUS, or FFR, the model can automatically learn and internalize the “functional knowledge” implicitly contained in multimodal imaging and hemodynamic parameters from routine invasive CAG images. In current interventional workflows for coronary artery disease, when CAG shows an intermediate lesion, clinicians often must rely on experience or further apply intracoronary imaging or FFR to clarify the lesion’s functional significance. Although these modalities can precisely assess vessel lumen morphology, plaque burden, stent apposition and expansion, and even detect microstructural abnormalities such as dissections, they suffer from clear limitations: long procedure time, high cost, operator dependence, and additional risks associated with guidewire manipulation and potential complications. Consequently, not all patients with intermediate lesions can undergo these assessments in practice, leaving some patients under- or over-treated due to insufficient diagnostic information. The development of artificial intelligence offers a new opportunity to address this challenge. Deep learning models, trained on a large number of CAG images, can automatically extract latent anatomic and hemodynamic features and predict the functional significance of lesions—thus providing a rapid and resource-efficient preliminary stratification tool for intermediate lesions that avoids the additional burden of adjunctive invasive examinations beyond the index catheterization. Our proposed model significantly enhances diagnostic efficiency and consistency, reduces bias arising from operator experience variability, and holds promise to serve as an “intelligent sentinel” in the catheterization laboratory—improving workflow while reducing unnecessary OCT, IVUS, or FFR procedures and producing substantial savings in cost and time. This work not only overcomes the previous limitation of separating morphological imaging and functional evaluation, but also offers a novel technical pathway for intelligent functional assessment of coronary lesions. Thus, the findings of this study carry important innovation value and great potential for the model’s clinical translation in facilitating AI-assisted precision diagnosis and treatment of coronary heart disease.

The clinical positioning of the proposed model relative to angiography-derived FFR (QFR/iFR) and CT-derived FFR warrants explicit discussion. QFR, computed from standard angiographic acquisitions using computational fluid dynamics, has demonstrated diagnostic accuracy comparable to wire-based FFR while eliminating adenosine administration^31^. CT-derived FFR (HeartFlow FFRCT) offers pre-procedural functional assessment without catheterization but requires dedicated post-processing infrastructure and specialized acquisition protocols^32^. The proposed model operates directly on standard CAG images already acquired during catheterization, requiring no additional pharmacological agents, no specialized acquisition modifications, and no external computational fluid dynamics platform—attributes that may facilitate adoption in resource-limited or time-constrained settings. Its external validation AUC of 0.897 is comparable to published QFR benchmarks, suggesting potential clinical complementarity rather than simple substitution. However, direct head-to-head comparison with QFR and FFRCT in the same patient cohort with shared ground-truth FFR measurements is necessary to definitively establish relative utility, and represents a priority direction for future prospective validation.

The superior performance of Improved_EfficientNet over alternative architectures reflects a coherent synergy among its core design choices. At the architectural level, three complementary factors collectively account for its representational advantage: EfficientNet’s compound scaling strategy simultaneously optimizes network depth, width, and input resolution, yielding a favorable capacity-to-parameter ratio that constrains overfitting in moderate-sized medical imaging datasets; CBAM integration dynamically suppresses irrelevant background features ubiquitous in angiographic images—including catheter artifacts and non-target vessels—while selectively amplifying lesion-specific spatial and channel signals; and Focal Loss with class weighting addresses the near-balanced class distribution, improving recall without sacrificing specificity. It is noteworthy that most existing AI studies on coronary artery lesions have primarily focused on morphological features of imaging—such as quantifying stenosis severity^33,34^, MLA^35^, or calcium burden^36–38^—or have been limited to lesion detection and localization tasks. Although these approaches have advanced structural recognition, they lack modeling of the functional significance of lesions and the clinical decision-making process, making them insufficient to directly guide key therapeutic decisions such as stent implantation. The present model addresses this gap through three clinically oriented design principles that collectively bridge morphological imaging and functional decision-making: a clinically grounded learning objective that incorporates OCT/IVUS/FFR-derived assessments and actual treatment outcomes as supervision labels, enabling the network to internalize multimodal representations aligned with real-world clinical reasoning; attention-guided feature localization via CBAM, directing the model’s focus toward stenotic regions and zones of local hemodynamic variation to capture subtle coronary structural and functional differences with high fidelity; and quantitative interpretability validation through Grad-CAM, which confirmed that the model’s attention regions exhibit high spatial concordance with true stenotic segments, achieving strong expert-blinded evaluation scores and demonstrating consistency between model focus and interventional cardiologists’ diagnostic logic—effectively mitigating the “black box” concern in medical imaging AI. These properties collectively underpin the model’s strong and consistent performance across both internal and external validation cohorts, surpassing all comparator architectures—including DenseNet121, EfficientNet, InceptionV3, MobileNetV2, ResNet50, Swin-T, and VGG11—as well as alternative multimodal fusion strategies including Cross Modal Attention, GMU, and Late Fusion.

Moreover, the conceptual approach of this study aligns well with the ongoing cross-disciplinary trend of AI applications in other medical domains. In recent years, AI techniques have shown remarkable potential in oncology, neuroimaging, and musculoskeletal imaging. For instance, in oncology, deep learning models have been used to predict molecular subtypes, drug sensitivity, and prognostic risk in breast cancer^39,40^, lung cancer^41^, and gliomas^42^—providing vital support for precision therapy and individualized decisions. In orthopedic imaging, AI combined with radiomics analysis has been widely applied to intelligent monitoring of scoliosis^43^, three-dimensional posture reconstruction, and surgical risk assessment—realizing intelligent and dynamic disease management. In neurological disorders, deep learning models based on MRI have been able to detect early changes of Alzheimer’s disease^44^ or predict the progression trajectory of Parkinson’s disease. These cross-disciplinary applications fully demonstrate AI’s ability to automatically learn high-dimensional biological patterns from imaging data, integrate multimodal information, and gradually internalize clinical reasoning logic. Our study is the first to systematically apply this paradigm to functional assessment in coronary disease, thereby filling the critical gap from “structural recognition” to “functional inference” and embodying an innovative path for AI-empowered precision cardiovascular imaging with translational potential.

An important consideration of the present study is that, in a subset of patients, outcome labels were derived from actual clinical treatment decisions (i.e., stent implantation). This may raise concerns regarding potential circularity, particularly given the well-established evidence that angiography alone is an imperfect surrogate for the functional significance of coronary stenosis and that guideline-recommended techniques such as FFR, OCT, and IVUS were developed precisely to address this limitation. Importantly, however, a substantial proportion of cases in this cohort were labeled using objective reference standards, which anchors the model to established physiological or structural criteria and meaningfully mitigates the risk of pure decision replication. Accordingly, the present study does not aim to suggest that angiographic images alone are sufficient to determine lesion-level ischemic significance or to challenge existing guideline recommendations. Instead, the proposed model is designed to characterize and standardize real-world interventional decision-making in clinical settings where comprehensive physiological or intravascular assessment is unavailable, deferred, or selectively applied. In this context, the model should be viewed as a decision-support tool that reflects prevailing clinical practice under practical constraints, rather than as a replacement for physiological assessment or a surrogate for causally determining lesion significance. Nevertheless, we acknowledge that treatment decisions, even when informed by multiple data sources, remain subject to clinical judgment and external factors, and future studies incorporating uniformly applied physiological endpoints and prospective validation will be required to further disentangle decision modeling from causal assessment of coronary lesion significance.

Additionally, the cross-validation AUC of 0.997, while unusually high, is attributable to four convergent contextual factors rather than data leakage. First, this is a binary classification task, and published deep learning models for binary cardiac imaging classification have reported comparable values (e.g., echocardiographic view classification AUC = 0.997–0.998; cardiac disease subtype identification AUC = 0.985–0.999), confirming such performance is mechanistically plausible. Second, the cohort was restricted to intermediate lesions only (50–70% stenosis), a pre-selected population with reduced phenotypic heterogeneity and a more learnable decision boundary than general CAD populations. Third, the reference standard was derived predominantly from objective FFR and OCT/IVUS assessments, minimizing label noise—a recognized driver of inflated in-sample performance in weakly supervised settings. Fourth, and most critically, the AUC reduction from CV (0.997) to external validation (0.897) is itself the strongest evidence against data leakage: genuine leakage produces inflated performance that persists across settings, whereas the observed 0.100-point degradation authentically reflects inter-institutional domain shift in acquisition protocols and patient case-mix. Notably, the external validation AUC of 0.897 remains within the excellent discrimination range by conventional criteria, and a performance decrement of this magnitude across independent multi-institutional cohorts is consistent with published benchmarks for cross-center deep learning validation in cardiac imaging, further supporting the interpretation that the observed degradation reflects expected domain shift rather than any fundamental limitation in generalizability.

Like many early-phase AI studies in interventional cardiology, the present study does not yet incorporate post-procedural clinical outcome data. The model predicts the binary stent implantation decision rather than downstream clinical events such as TLR, MACE, or physiological recovery metrics (e.g., post-PCI FFR). This distinction carries significant clinical implications: a model trained on historical treatment decisions may internalize prevailing clinical practice patterns—including their known guideline-adherence variability—rather than identifying the subset of patients who would derive genuine prognostic benefit from revascularization. In settings where evidence-practice gaps exist (e.g., overutilization or underutilization of stenting), the model may perpetuate rather than correct these patterns. For the model to be recommended for autonomous clinical implementation, validation against hard clinical endpoints (2-year MACE, angina burden, quality-of-life metrics) would be required. We wish to be explicit that the current system is designed as a decision-support tool to assist, not replace, physician judgment, and that its clinical value should be interpreted in this context. Addressing these limitations will require a prospective multicenter outcomes-based validation study incorporating standardized FFR measurement, 2-year clinical follow-up for all enrolled patients, and deployment across additional external centers spanning different geographic regions and healthcare systems—an initiative currently in the planning phase that will simultaneously provide the most rigorous assessment of cross-institutional clinical performance and serve as the basis for a subsequent publication.

Furthermore, we acknowledge that the reliance on single-frame 2D angiographic images introduces inherent geometric limitations, including projection bias, vessel foreshortening, and overlap. 3D coronary reconstruction or multi-projection 3D-CAG inputs could potentially provide a more comprehensive assessment of lesion morphology and further reduce the subjective bias associated with manual frame selection. While our sensitivity analysis demonstrated high consistency (ICC = 0.89) among independent observers using the current 2D approach, and the model achieved strong discriminative performance across both internal and external validation cohorts, transitioning to a 3D-based or multi-frame volumetric input remains a priority for future iterations, as such advancements would likely further enhance robustness in complex anatomical scenarios involving vessel foreshortening or significant overlap. That said, the computational demands of 3D reconstruction and volumetric deep learning are substantially higher than those of 2D image-based inference—requiring dedicated GPU infrastructure, larger memory capacity, and longer processing pipelines that may be prohibitive in resource-limited settings such as community hospitals or healthcare systems in developing regions where high-end hardware and the associated economic investment may not be readily available.

In this respect, the 2D-based design of the present model represents not only a practical compromise but an accessibility-oriented design: by operating directly on standard single-frame CAG images already acquired during routine catheterization, the model is deployable without additional infrastructure or workflow modification, broadening its potential reach to precisely those clinical environments where adjunctive physiological assessment is least accessible. Nevertheless, we recognize that 3D-based approaches offer inherent geometric advantages, and future iterations will pursue two complementary strategies to progressively close this gap without imposing prohibitive hardware requirements: first, algorithmic optimization aimed at reducing the computational overhead of volumetric inference through lightweight architecture design and model compression techniques; and second, multi-projection 3D reconstruction from standard multi-angle 2D angiographic acquisitions—a mathematically principled approach that leverages geometric consistency across viewing angles to infer three-dimensional coronary structure without dedicated 3D imaging hardware, analogous to established methods in structure-from-motion and epipolar geometry. Taken together, the present study introduces a validated, interpretable, and broadly deployable deep learning framework that bridges the longstanding gap between morphological coronary imaging and functional clinical decision-making—offering a resource-efficient, clinician-assistive tool with meaningful potential for improving the consistency and equity of interventional care across diverse healthcare settings.

## Methods

### Study population

This study was designed as a retrospective, multicenter cohort study conducted between 2016 and 2025. Patients were identified from the catheterization laboratory databases of three tertiary academic medical centers in China (The First Affiliated Hospital of Nanjing Medical University, Ningxia Hui Autonomous Region Hospital of Traditional Chinese Medicine, and The First Affiliated Hospital of Anhui Medical University).

The study cohort was intentionally restricted to patients with angiographically intermediate coronary lesions (50–70% diameter stenosis on CAG, estimated visually or by QCA) who underwent additional intravascular or physiological assessment due to diagnostic uncertainty. The cohort included patients across the full spectrum of clinical presentations, including stable CAD (*n* = 753, 58.0%), unstable angina (*n* = 285, 22.0%), NSTEMI (*n* = 182, 14.0%), and STEMI (*n* = 78, 6.0%). In STEMI patients, adjunctive functional or structural assessment of a non-culprit intermediate lesion identified during the index procedure was performed at the discretion of the treating physician and served as the basis for label assignment in these cases. Accordingly, patients who received at least one further evaluation modality (FFR, OCT, or IVUS) during or after the index CAG were screened. Patients whose treatment decisions were based solely on angiography were excluded, as the absence of an objective reference standard precluded reliable label assignment. A total of 1,298 patients met the eligibility criteria and were included in the final analysis. Among these patients, 756 (58.2%) underwent FFR assessment, 312 (24.0%) underwent IVUS, and 230 (17.8%) underwent OCT as the primary reference modality for treatment decision-making. Note that some patients underwent more than one adjunctive modality during the index procedure; in such cases, the modality with the highest hierarchical priority (FFR > OCT/IVUS) was designated as the primary reference standard, and the patient was counted under that modality only. Therefore, these figures represent the number of patients whose final outcome label was derived from each respective modality, rather than the total number of patients who underwent each assessment.

**Fig. 7:**
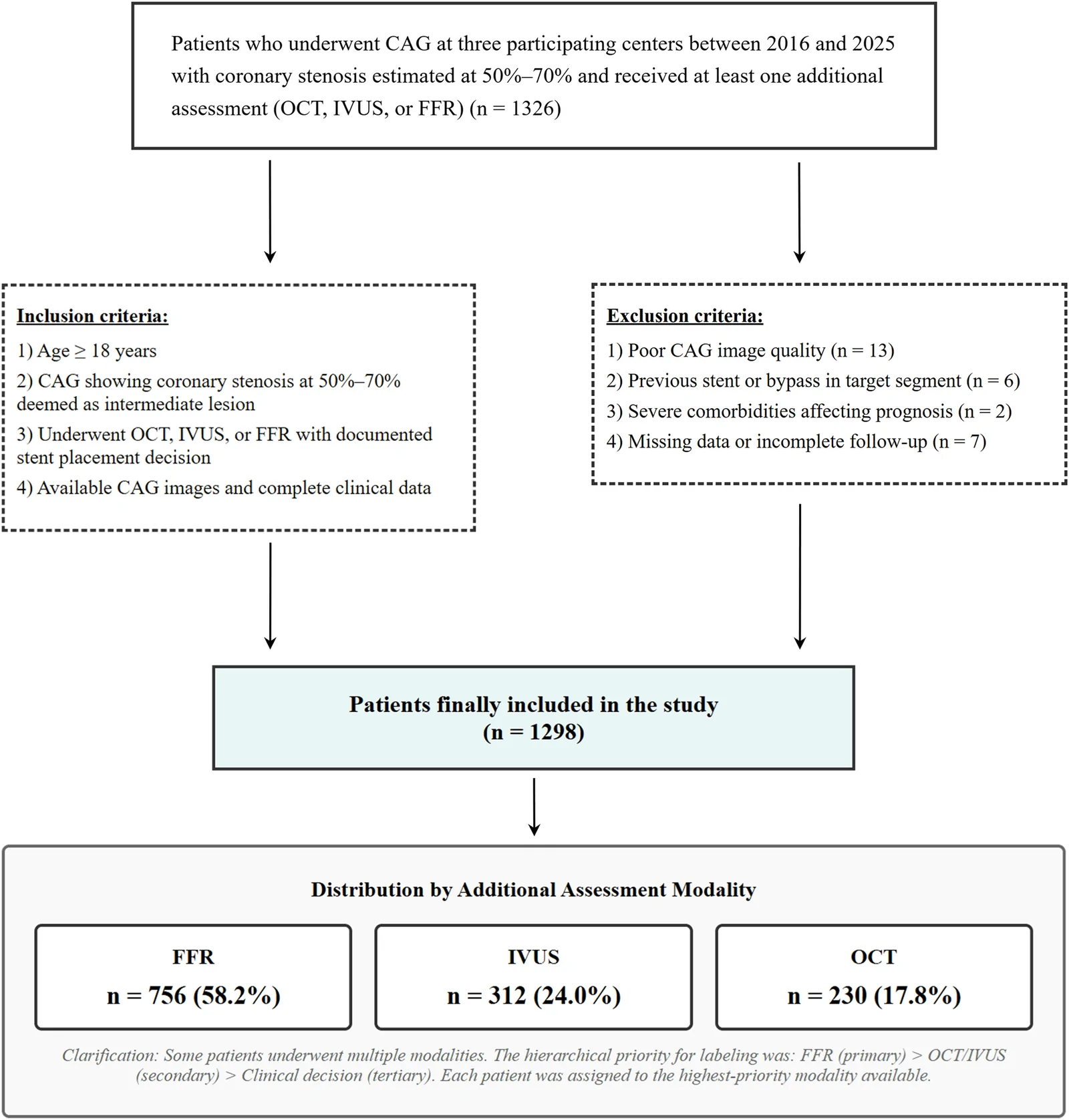
Patient inclusion and exclusion criteria for study enrollment with distribution by assessment modality. The final cohort was stratified by primary reference modality: FFR (*n* = 756, 58.2%), IVUS (*n* = 312, 24.0%), and OCT (*n* = 230, 17.8%). CAG coronary angiography, OCT optical coherence tomography, IVUS intravascular ultrasound, FFR fractional flow reserve.

The inclusion and exclusion criteria are summarized in Fig. 7. Inclusion criteria were: (1) age ≥18 years; (2) at least one intermediate coronary lesion (50–70% stenosis on CAG); (3) availability of at least one additional intravascular or physiological assessment (FFR, OCT, or IVUS) for the target lesion with a definitive stent decision; and (4) complete CAG images and clinical data. Exclusion criteria included insufficient image quality, prior revascularization of the target segment, severe comorbidities affecting prognosis or imaging quality, and missing outcome or follow-up data.

This study was performed in accordance with the Declaration of Helsinki (1975) and its subsequent revisions. The study protocol was centrally approved by the Ethics Committee of The First Affiliated Hospital with Nanjing Medical University (Lead Center; Approval No. 2022-SR-529). The Ethics Committees of the Ningxia Hui Autonomous Region Hospital of Traditional Chinese Medicine and The First Affiliated Hospital of Anhui Medical University formally reviewed and accepted the central ethical approval from the lead institution. Due to the retrospective nature of this study and the use of fully de-identified clinical and imaging data, the requirement for informed consent was waived by the respective ethics committees of all participating institutions. All retrospective data were anonymized prior to analysis, and all DICOM data were de-identified by removing or replacing patient identifiers (such as names and admission numbers) to comply with hospital information security protocols. To ensure objectivity in labeling and model training, OCT/IVUS/FFR results and stent implantation decisions were independently reviewed by at least two cardiovascular specialists or interventional radiologists, with any discrepancies resolved by a senior expert adjudicator.

### Data collection and annotation

In this study, CAG images were used as model inputs. The outcome variable was the binary clinical decision regarding stent implantation, defined according to a hierarchical multimodal reference standard. Specifically, FFR served as the primary physiological reference standard. For lesions in which FFR was not available, intravascular imaging modalities—including OCT or IVUS—served as secondary structural reference standards. All patients included in the final analytic cohort had at least one objective reference modality for the target lesion. The defined outcome therefore, reflects real-world interventional decision-making informed by multimodal diagnostic assessment rather than post-procedural physiological outcomes such as residual FFR, target lesion revascularization, or major adverse cardiac events (MACE). During label construction, patients who ultimately underwent stent implantation or other interventional treatments were assigned to the positive set (Need Stent), whereas patients who did not receive such interventions were assigned to the negative set (No Stent). The label definition and processing workflow are summarized as follows.

The outcome label “Need Stent” was defined at the patient level based on a single target lesion—the clinically relevant lesion that directly guided the interventional decision. When multiple intermediate lesions were present, only the lesion evaluated by FFR, OCT, or IVUS that determined stent implantation was labeled. Each patient received one binary label (“Need Stent” or “No Stent”) without aggregation across multiple lesions. To minimize heterogeneity and address differences in diagnostic principles, a hierarchical priority-based framework was established.

FFR served as the primary physiological reference standard with highest priority. Per clinical guidelines, FFR ≤ 0.80 indicated functionally significant ischemia (Need Stent), while FFR > 0.80 indicated non-significance (No Stent). When available for the target lesion, FFR exclusively determined the label. OCT and IVUS were secondary structure-based standards used only when FFR was absent. Structural positivity was operationally defined using established evidence-based thresholds: MLA < 3.0 mm² for proximal LAD lesions or <2.75 mm² for mid-LAD lesions proximal to the second diagonal branch^45^; and for non-LAD vessels, vessel size-adjusted thresholds of <2.4 mm² (reference diameter <3.0 mm), <2.7 mm² (reference diameter 3.0–3.5 mm), or <3.6 mm² (reference diameter >3.5 mm) per the 2018 ESC/EACTS Guidelines on Myocardial Revascularization; plaque burden ≥ 70% on IVUS^46^; or presence of high-risk plaque morphology on OCT including thin-cap fibroatheroma with fibrous cap thickness <65 μm, plaque rupture, or plaque erosion with overlying thrombus^47^. Cases categorized as indeterminate on OCT/IVUS where no FFR was available were excluded from the final analysis to preserve label integrity^48,49^. To ensure the highest label integrity, a post-hoc audit was performed for all included cases. Any instance where the final treatment decision deviated from these predefined objective reference standards (e.g., stenting performed in the absence of functional ischemia or significant structural obstruction as defined above) was considered non-adherent and subsequently excluded from the study cohort. This rigorous filtering ensures that the training labels reflect evidence-based clinical standards rather than idiosyncratic operator variability. All OCT/IVUS measurements underwent standardized re-evaluation by experienced specialists to reduce variability.

Clinical treatment decisions served as a tertiary inclusion criterion to identify eligible cases when neither FFR nor structural imaging had been applied to the target lesion; however, the definitive outcome label for all such cases was assigned according to the highest-evidential-weight modality available among FFR, OCT, and IVUS, ensuring consistent label integrity across the three classification groups. This tertiary label source was restricted to cases with documented longitudinal follow-up (≥12 months event-free survival or target lesion revascularization) to minimize label circularity. The potential implications of this approach—including the risk that the model may partially reflect existing clinical decision patterns rather than purely physiological ischemia determinants—are discussed in the Limitations section.

The hierarchical framework (FFR > OCT/IVUS >adjudicated treatment decision) ensured each label reflected a single patient-level decision without mixing reference modalities. Consequently, although a subset of patients received more than one adjunctive assessment, each patient contributed exactly one binary label to the dataset, and the modality-specific counts reported in Section “Baseline characteristics” reflect label source attribution rather than examination frequency. The predominance of FFR-based labels minimizes label circularity concerns while maintaining clinical representativeness. The potential impact on model generalization is discussed as a limitation: the model may partially reflect existing clinical decision patterns, potentially affecting performance in settings with substantially different practice patterns. Future studies incorporating prospective FFR validation for all cases or outcomes-based labels (e.g., major adverse cardiac events) may further address this limitation and enhance the model’s ability to capture purely physiological ischemia determinants.

For quantitative parameters such as MLA obtained from OCT/IVUS, measurement protocols and equipment may vary between clinical teams at different centers. To minimize systematic bias, a standardized measurement procedure was implemented: all OCT/IVUS images were re-analyzed using the same software or standardized measurement steps (including identical pixel scaling and consistent cross-sectional selection rules), or independently re-measured in a blinded manner by two imaging specialists, with the average value used for analysis.

For subjective evaluations (e.g., severe calcification, unstable plaque), dual independent reviews were performed, with a senior expert resolving disagreements. Inter-rater agreement between the two imaging specialists was strong (Cohen’s kappa = 0.87). To further assess label reliability, a blinded re-adjudication of a random 10% subsample (*n* = 130) was performed by an external cardiologist from a non-participating institution; agreement with the primary labels was 94.6% (kappa = 0.89). OCT/IVUS interpretations were ultimately categorized as “structurally positive,” “structurally negative,” or “indeterminate,” and incorporated into the final labeling hierarchy.

To ensure robust model development using multi-center data while rigorously evaluating generalizability, we adopted a multi-center data partitioning strategy with strict separation to prevent data leakage. Specifically, to prevent data contamination: (1) all images from the same patient were strictly confined to a single fold and never appeared simultaneously in training and validation sets; (2) all preprocessing parameters (CLAHE settings, normalization statistics, data augmentation seeds) were derived solely from training-fold data and applied identically to validation folds; (3) no hyperparameter optimization was performed using external validation data; and (4) the external validation cohort was completely isolated from the entire model development pipeline. Patients from The First Affiliated Hospital with Nanjing Medical University and Ningxia Hui Autonomous Region Hospital of Traditional Chinese Medicine were combined to form the training cohort (total *n* = 980) for model development and internal evaluation. The First Affiliated Hospital of Anhui Medical University served as an independent external validation cohort (*n* = 318) to assess model performance on truly heterogeneous, unseen data from a different institution.

All analyses were conducted at the patient level. Within the training cohort, patients were randomly divided using stratified 5-fold cross-validation, with stratification based on the binary outcome label (Need Stent / No Stent) and center affiliation to maintain balanced class distribution and proportional representation of each contributing center across all folds. In each iteration, the model was trained on four folds (approximately 80% of the training cohort), while the remaining fold (approximately 20%) was used for internal validation, including hyperparameter tuning and early stopping. This process was repeated five times, with each fold serving once as the validation set. Final internal performance metrics were reported as the mean ± standard deviation across the five cross-validation folds.

The external validation cohort, consisting exclusively of patients from The First Affiliated Hospital of Anhui Medical University, was completely isolated from the entire model development pipeline, including preprocessing, feature extraction, and hyperparameter optimization. All preprocessing parameters were estimated exclusively from the training data and subsequently applied to the external validation cohort without recalibration. This design enabled an unbiased assessment of real-world generalizability across institutional, equipment, and population differences.

Overall model performance was summarized using the averaged results from the 5-fold internal cross-validation, together with the independent performance observed on the external validation cohort.

### Image preprocessing

To ensure methodological reproducibility and robustness across different angiography systems, acquisition protocols, and patient characteristics, a standardized and fully specified preprocessing pipeline was implemented for all CAG images.

CAG data were originally acquired as cine loops containing multiple frames per projection. As this study focused on a single-frame, image-based deep learning model, a representative frame was selected for each target lesion to ensure optimal image quality and diagnostic clarity.

Frame selection was performed using a semi-automated expert-guided strategy to balance reproducibility with clinical realism. Specifically, from each cine loop, the frame corresponding to peak contrast opacification of the target vessel segment and minimal motion artifact was identified. Peak contrast was operationally defined as the frame exhibiting maximal and homogeneous contrast filling of the target vessel lumen while preserving clear delineation of lesion boundaries. Selection was performed by an experienced interventional cardiologist (≥5 years of experience), blinded to the final ischemia label, with subsequent independent verification by a second reviewer. To minimize site-specific selection bias, the three cardiologists involved in the sensitivity analysis were each affiliated with a different participating institution (one from The First Affiliated Hospital of Nanjing Medical University, one from Ningxia Hui Autonomous Region Hospital of Traditional Chinese Medicine, and one from The First Affiliated Hospital of Anhui Medical University). In cases of inter-rater disagreement (occurring in <5% of cases), consensus was reached through joint review and discussion.

To assess the potential impact of frame selection variability on model performance, we conducted a sensitivity analysis in a subset of 50 randomly selected cases. Three independent cardiologists each selected optimal frames from the same cine loops following the predefined criteria. Model predictions showed high consistency across different frame selections (intra-class correlation coefficient = 0.89, 95% CI: 0.85–0.92), suggesting that the model is relatively robust to minor frame-to-frame variations when images meet quality standards. Nevertheless, we recognize that frame variability remains a potential source of uncertainty, particularly in lesions with dynamic flow characteristics or suboptimal image quality.

Selected frames were exported from DICOM format and converted to lossless PNG or TIFF images to avoid compression artifacts. All images were fully de-identified by removing patient identifiers, timestamps, and embedded overlays.

To focus the model on lesion-relevant regions while preserving sufficient anatomical context, images were manually or semi-automatically cropped to include the target vessel segment and adjacent proximal and distal regions. Cropping was performed such that the lesion was centered within the field of view whenever possible, while excluding irrelevant background regions (e.g., catheter tips, collimator edges). The resulting region of interest typically covered approximately 60–80% of the original frame area, depending on vessel orientation and projection. It is acknowledged that the current cropping procedure relies on manual or semi-automated operator input. To address this scalability limitation, an integrated vessel segmentation preprocessing module based on a U-Net architecture trained on annotated angiographic frames is currently under development to enable fully automated region-of-interest extraction, which will be incorporated into the clinical software system (version 2.0).

To enhance vessel–background contrast and improve visualization of subtle lumen narrowing, CLAHE was applied. CLAHE was performed on grayscale images using fixed parameters across all samples to ensure consistency: a tile grid size of 8×8 and a clip limit of 2.0. These parameters were selected empirically to enhance local contrast without amplifying noise or introducing artificial edges.

Following CLAHE, pixel intensities were normalized to the range [0, 1]. For models initialized with ImageNet-pretrained weights, standard mean and standard deviation normalization were applied accordingly.

All images were resized to a fixed spatial resolution to match the input requirements of the backbone networks. Specifically, images were resized to 224 × 224 pixels using bilinear interpolation. To preserve fine-grained structural details, contrast enhancement was applied prior to resizing.

During training, data augmentation was employed to improve generalizability and simulate real-world variability in angiographic acquisition. Augmentation operations included random resized cropping (scale range: 0.7–1.0), random rotation (±15°), horizontal and vertical flipping (probability = 0.5), random brightness and contrast adjustment, and mild Gaussian noise. These augmentations were applied on-the-fly during training only. During validation and testing, deterministic preprocessing (center crop, resizing, and normalization) was applied without augmentation.

All preprocessing steps, including frame selection criteria, cropping strategy, CLAHE parameters, resizing resolution, and augmentation settings, were fixed prior to model training and applied identically across training, internal validation, and external validation cohorts. This standardized pipeline facilitates reproducibility and supports potential clinical deployment. While the current study relies on single-frame inputs, future extensions will investigate automated frame selection, multi-frame fusion, and temporal modeling to further enhance robustness and reduce frame dependence.

### Model architecture

EfficientNet was adopted as the backbone due to its balance between parameter efficiency, computational cost, and representational power^50–52^. EfficientNet employs compound scaling across depth, width, and resolution, allowing smaller models to achieve high accuracy under limited computational resources^53,54^. Pretrained weights on ImageNet were used for transfer learning, followed by fine-tuning on CAG images. High-level semantic feature maps were extracted for subsequent modules to ensure abstract yet spatially informative representations. Specifically, the stem convolution layer and the first two Mobile Inverted Bottleneck Convolution (MBConv) blocks of EfficientNet-B0 (stages 0–1, representing approximately the first 30% of network depth by parameter count) were frozen during the initial 20 training epochs to preserve low-level feature representations and stabilize convergence. All remaining layers—including MBConv blocks 3–7, the top convolution, and the classification head—were subsequently unfrozen for full end-to-end fine-tuning at a reduced learning rate of 1 × 10⁻⁵. The transition was triggered when training loss improvement fell below 0.001 for ≥3 consecutive epochs.

The original CAG image input to the model is denoted as ${\boldsymbol{X}}$, and its high-level feature map is represented as F, where $F\in {{\mathbb{R}}}^{C\times H\times W}$. To enhance the model’s responsiveness to local stenotic segments and regions of disturbed blood flow, a CBAM was embedded after the high-level feature map ${\boldsymbol{F}}$. CBAM is composed of a channel attention module ${{\boldsymbol{M}}}_{{\boldsymbol{c}}}(\cdot )$ and a spatial attention module ${{\boldsymbol{M}}}_{{\boldsymbol{s}}}(\cdot )$ in a sequential manner. Its operation can be described as follows: first, the channel-recalibrated feature map is computed as:

1 $${{\boldsymbol{F}}}^{{\prime} }={{\boldsymbol{M}}}_{{\boldsymbol{c}}}\left({\boldsymbol{F}}\right)\bigotimes {\boldsymbol{F}}$$

Then the spatially recalibrated feature map is computed as:

2 $${{\boldsymbol{F}}}^{{\prime} {\prime} }={{\boldsymbol{M}}}_{{\boldsymbol{s}}}\left({{\boldsymbol{F}}}^{{\prime} }\right)\bigotimes {{\boldsymbol{F}}}^{{\prime} }$$

where ${\boldsymbol{\otimes }}$ denotes element-wise multiplication.

The calculation of channel attention is given by:

3 $${{\boldsymbol{M}}}_{{\boldsymbol{c}}}\left({\boldsymbol{F}}\right)={\boldsymbol{\sigma }}\left({\boldsymbol{MLP}}\left({\boldsymbol{GAP}}\left({\boldsymbol{F}}\right)\right)+{\boldsymbol{MLP}}\left({\boldsymbol{GMP}}\left({\boldsymbol{F}}\right)\right)\right)$$

where global average pooling (GAP) and global max pooling (GMP) are applied along the spatial dimensions of ${\boldsymbol{F}}$ to generate vectors ${{\boldsymbol{z}}}_{{\boldsymbol{avg}},}{{\boldsymbol{z}}}_{{\boldsymbol{m}}{\boldsymbol{a}}{\boldsymbol{x}}}{\boldsymbol{\in }}{{\rm{{\mathbb{R}}}}}^{{\boldsymbol{C}}}$, respectively, the MLP is a shared two-layer fully connected network (the first layer reduces the dimension to $\frac{{\boldsymbol{C}}}{{\boldsymbol{r}}}$, and the second restores it back to ${\boldsymbol{C}}$; in this study, ${\boldsymbol{r}}=16$). ${\boldsymbol{\sigma }}$ denotes the Sigmoid activation. The output ${{\boldsymbol{M}}}_{{\boldsymbol{c}}}\left({\boldsymbol{F}}\right)$ has the shape ${\boldsymbol{C}}\times {\boldsymbol{1}}\times {\boldsymbol{1}}$, which is then broadcast and multiplied with ${\boldsymbol{F}}$ to achieve channel-wise weighting.

The computation of spatial attention is formulated as:

4 $${{\boldsymbol{M}}}_{{\boldsymbol{s}}}\left({{\boldsymbol{F}}}^{{\prime} }\right)=\sigma \left({{\boldsymbol{Conv}}}_{{\bf{7}}\times {\bf{7}}}\left(\left[\bar{{{\boldsymbol{F}}}^{{\prime} }};\widetilde{{{\boldsymbol{F}}}^{{\prime} }}\right]\right)\right)$$

5 $$\bar{{{\boldsymbol{F}}}^{{\prime} }}={{\boldsymbol{Mean}}}_{{\boldsymbol{ch}}}\left({{\boldsymbol{F}}}^{{\prime} }\right),\bar{{{\boldsymbol{F}}}^{{\prime} }}\in {{\mathbb{R}}}^{{\bf{1}}\times {\boldsymbol{H}}\times {\boldsymbol{W}}}$$

6 $$\widetilde{{{\boldsymbol{F}}}^{{\prime} }}={{\boldsymbol{Max}}}_{{\boldsymbol{ch}}}\left({{\boldsymbol{F}}}^{{\prime} }\right),\widetilde{{{\boldsymbol{F}}}^{{\prime} }}\in {{\mathbb{R}}}^{{\bf{1}}\times {\boldsymbol{H}}\times {\boldsymbol{W}}}$$

Here, $\bar{{{\boldsymbol{F}}}^{{\boldsymbol{{\prime} }}}}$ and $\widetilde{{{\boldsymbol{F}}}^{{\boldsymbol{{\prime} }}}}$ represent the spatial maps obtained by averaging and taking the maximum along the channel dimension, respectively. The notation $\left[\bar{{{\boldsymbol{F}}}^{{\prime} }};\widetilde{{{\boldsymbol{F}}}^{{\boldsymbol{{\prime} }}}}\right]$ denotes channel-wise concatenation, and ${{\boldsymbol{Conv}}}_{{\boldsymbol{7}}\times {\boldsymbol{7}}}$ indicates a convolution with a 7×7 kernel. Finally, a Sigmoid activation is applied to generate the spatial attention map ${{\boldsymbol{M}}}_{{\boldsymbol{s}}}\left({{\boldsymbol{F}}}^{{\prime} }\right)$.

The sequential composition of CBAM enables the model to identify *which channels* (i.e., lesion-related features) are important and *which spatial locations* (i.e., lesion sites) are critical, thereby amplifying localized but clinically relevant signals.

The attention-enhanced feature map is compressed into a spatially invariant feature vector via adaptive average pooling, which is then fed into the classification head. The classification head consists of two fully connected layers: the first serves as an intermediate representation layer (512 neurons, followed by ReLU activation and Dropout regularization), and the second outputs the final classification logits (binary classification in this study). This design allows the classifier to retain sufficient nonlinear expressiveness while keeping the parameter scale manageable, and Dropout helps mitigate overfitting.

In addition to the classification output, we retain the intermediate feature vector before classification as a general-purpose representation for subsequent PCA visualization analyses, clustering, and uncertainty estimation. This vector can also serve as an input for multimodal fusion with clinical variables or other imaging modalities, thereby facilitating future model expansion and clinical integration.

To ensure interpretability of predictions and facilitate clinical validation, we adopted Grad-CAM to generate class-specific activation heatmaps and quantitatively evaluate them. Let the ${\boldsymbol{k}}$-th channel feature map of the target convolutional layer be denoted as ${{\boldsymbol{A}}}^{{\boldsymbol{k}}}{\boldsymbol{\in }}{{\mathbb{R}}}^{{\boldsymbol{H}}\times {\boldsymbol{W}}}$, and the unnormalized score for the target class ${\boldsymbol{c}}$ as ${{\boldsymbol{y}}}^{{\boldsymbol{c}}}$. Grad-CAM computes gradient-based weighting coefficients as follows:

7 $${{\boldsymbol{\alpha }}}_{{\boldsymbol{k}}}^{{\boldsymbol{c}}}=\frac{{\bf{1}}}{{\boldsymbol{Z}}}\mathop{\sum }\limits_{{\boldsymbol{i}}}\mathop{\sum }\limits_{{\boldsymbol{j}}}\frac{{\boldsymbol{\partial }}{{\boldsymbol{y}}}^{{\boldsymbol{c}}}}{{\boldsymbol{\partial }}{{\boldsymbol{A}}}_{{\boldsymbol{i}}{\boldsymbol{j}}}^{{\boldsymbol{k}}}}$$

Where ${\boldsymbol{Z}}={\boldsymbol{H}}\times {\boldsymbol{W}}$ is the total number of pixels. The class-discriminative activation map is then constructed as:

8 $${{\boldsymbol{L}}}_{{\bf{Grad}}-{\bf{CAM}}}^{{\boldsymbol{c}}}={\boldsymbol{ReLU}}\left(\mathop{\sum }\limits_{{\boldsymbol{k}}}{{\boldsymbol{\alpha }}}_{{\boldsymbol{k}}}^{{\boldsymbol{c}}}{{\boldsymbol{A}}}^{{\boldsymbol{k}}}\right)$$

The resulting heatmap is upsampled bilinearly to the input resolution, min-max normalized to the range $[\mathrm{0,1}]$, and overlaid onto the original image to visualize the regions attended by the model. To mitigate instability caused by single-gradient noise, gradient smoothing (mean filtering or averaging across multiple forward-backward passes) or ensemble averaging across multiple models can be employed.

For quantitative evaluation of explainability, expert blinded assessment and Intersection-over-Union (IoU) metrics were used. If the expert-annotated lesion region is denoted as ${\bf{G}}$ and the binarized Grad-CAM region as ${\boldsymbol{S}}$, IoU is defined as:

9 $${\bf{IoU}}=\frac{\left|{\boldsymbol{G}}\cap {\boldsymbol{S}}\right|}{\left|{\boldsymbol{G}}\cup {\boldsymbol{S}}\right|}$$

We further computed the mean IoU and the “hit rate” (the proportion of heatmap centers falling within expert-annotated regions). Additionally, by collecting expert scores (e.g., 0–2 scale: 0 = no agreement, 1 = partial agreement, 2 = complete agreement), we quantitatively evaluated the consistency between heatmaps and physician interpretations, thereby demonstrating the contribution of the attention module to model interpretability and clinical relevance.

### Training and optimization

To achieve robust and reproducible model performance, a standardized training and validation pipeline was implemented. Five-fold cross-validation was performed at the patient level to evaluate model performance and prevent data leakage. All images from the same patient were strictly confined to a single fold and never appeared simultaneously in training and validation sets.

Model training was conducted for a maximum of 100 epochs with a batch size of 32. The AdamW optimizer was used with an initial learning rate of 1 × 10⁻⁴, and cosine annealing learning rate scheduling was applied to promote stable convergence. Focal Loss (*γ* = 2.0, *α* = 0.25) was adopted to address class imbalance, in combination with class weighting and oversampling. To mitigate label noise, label smoothing and noise-robust strategies such as mixup augmentation were employed, and uncertain-label cases were handled using soft labels or weak supervision.

Regularization strategies included dropout (*p* ≈ 0.4), L2 weight decay, and extensive data augmentation. Early stopping was applied to prevent overfitting: training was terminated if the validation loss failed to improve for more than 10 consecutive epochs, and the model achieving the best validation performance within each fold was retained. Mixed-precision training was used to reduce memory consumption and accelerate computation. In multi-GPU environments, Distributed Data Parallel was employed.

### Performance evaluation

To comprehensively assess model performance, we conducted a series of comparative experiments from three complementary perspectives: classification accuracy, model interpretability, and multimodal fusion effectiveness.

First, the proposed Improved EfficientNet was benchmarked against widely used deep learning architectures, including DenseNet121, EfficientNet, InceptionV3, MobileNetV2, ResNet50, Swin-T, and VGG11. Performance was evaluated using standard classification metrics (accuracy, sensitivity/recall, specificity, precision, and F1-score), along with confusion matrices and ROC curves with corresponding AUC values. Model interpretability was further examined using Grad-CAM heatmaps, which were qualitatively compared with expert-identified regions of interest. Quantitative explainability assessment was performed using intersection-over-union (IoU) and expert relevance scores (0–2). In addition, intermediate feature representations were projected into two-dimensional space using PCA to visualize class separability and discriminative feature learning.

Second, to evaluate the impact of multimodal fusion design, we compared four representative architectures integrating CAG images with structured clinical data: Cross-Modal Attention, Gated Multimodal Unit (GMU), Late Fusion, and the proposed model. All multimodal models were trained under an identical experimental protocol. Angiographic images were processed using the same EfficientNet-B0 backbone pretrained on ImageNet, while clinical variables were encoded via a three-layer multilayer perceptron with ReLU activation and dropout (*p* = 0.3). Fused features were passed to a shared two-layer classification head. Training settings, data preprocessing, augmentation strategies, dataset splits (64%/16%/20%), and random seeds were strictly standardized across models.

Beyond conventional performance metrics, multimodal models were further analyzed through predicted probability distribution analysis, threshold sensitivity evaluation, PCA-based feature visualization, and statistical comparison using McNemar’s tests with bootstrapped confidence intervals, providing a comprehensive assessment of prediction robustness and decision behavior.

## Supplementary information

Supplementary Information

## Data Availability

The datasets generated and/or analyzed during the current study are not publicly available due to patient privacy protection, confidentiality restrictions, and ethical regulations governing medical imaging data, but are available from the corresponding author on reasonable request. Access to de-identified patient data is subject to controlled access to ensure compliance with institutional review board (IRB) requirements, local regulatory and legal frameworks, and data sharing agreements between participating institutions. De-identified datasets may be made available to qualified researchers upon reasonable request to the corresponding author (Jiabao Liu: jiabaoliu@njmu.edu.cn). Requestors must provide: (a) a detailed research proposal outlining the purpose and planned analyses; (b) evidence of appropriate ethical approval from their institution; and (c) agreement to comply with a formal data use agreement that prohibits attempts to re-identify participants and restricts use to the approved research purpose only. Access requests will be evaluated within 30 days of submission.

## Code availability

The custom source code for the deep learning model architecture, including the attention-enhanced network design and Grad-CAM visualization implementation, is progressively being made available at https://github.com/XJSXJS888/Multimodal-CAG-Stent-Prediction-System.git. The repository includes model architecture specifications, training procedures, and evaluation scripts necessary to reproduce the main findings of this study, with complete code documentation and usage instructions provided. Trained model weights are subject to software copyright protection and will be made available to qualified researchers for academic research purposes upon reasonable request to the corresponding author, which should include a brief description of the intended use and confirmation of compliance with ethical requirements for clinical data analysis. The clinical software system (Coronary Angiography AI Diagnosis System, copyright registration No. 2025SR2285239) is proprietary software and not publicly available, though information about its architecture and functionality can be obtained by contacting the corresponding author. Information on the specific versions of Python-based deep learning frameworks and the primary parameters used to generate and analyze the datasets is detailed within the repository’s documentation and model specifications.
