# Supplementary material for: Deep learning predicts stent implantation in borderline coronary lesions from angiography
Source: NPJ Digit Med. 2026 May 22;9:739. doi: 10.1038/s41746-026-02779-z (PMC13624263; doi:10.1038/s41746-026-02779-z)
Supplement: Supplementary file 1 [file 41746_2026_2779_MOESM1_ESM.pdf]

Checklist for Artificial Intelligence in Medical Imaging (CLAIM): 2024 Update

Section / Topic	No.	Item	Page / Line	No	NA
**TITLE / ABSTRACT**					
	**1**	Identification as a study of AI methodology, specifying the category of technology used (e.g., deep learning)	**Page 1** (Title)		
**ABSTRACT**					
	**2**	Summary of study design, methods, results, and conclusions	**Page 2** (Abstract)		
**INTRODUCTION**					
	**3**	Scientific and/or clinical background, including the intended use and role of the AI approach	**Page 3** (Introduction)		
	**4**	Study aims, objectives, and hypotheses	**Pages 3-4** (Introduction)		
**METHODS**					
Study Design	**5**	Prospective or retrospective study	**Page 13** (Study Population)		
	**6**	Study goal	**Pages 14-15** (Data Collection and Annotation)		
Data	**7**	Data sources	**Pages 13-14** (Study Population)		
	**8**	Inclusion and exclusion criteria	**Page 14** (Study Population)		
	**9**	Data pre-processing	**Pages 17-18** (Image Preprocessing)		
	**10**	Selection of data subsets	**Page 16** (Data Partitioning Principles)		
	**11**	De-identification methods	**Pages 14 & 17** (Study Population & Image Extraction)		
	**12**	How missing data were handled	**Page 14** (Study Population -		

			Exclusion criteria)		
	**13**	Image acquisition protocol	**Page 17** (Frame Selection from Cine Angiography)		
Reference Standard	**14**	Definition of method(s) used to obtain reference standard	**Page 15** (Data Collection and Annotation)		
	**15**	Rationale for choosing the reference standard	**Page 15** (Data Collection and Annotation)		
	**16**	Source of reference standard annotations	**Page 14** (Study Population - Reviewers)		
	**17**	Annotation of test set	**Pages 15-16** (Data Collection and Annotation)		
	**18**	Measures of inter- and intra-rater variability of features described by the annotators	**Page 16** (Data Collection and Annotation)		
Data Partitions	**19**	How data were assigned to partitions	**Pages 16-17** (Data Partitioning Principles)		
	**20**	Level at which partitions are disjoint	**Page 16** (Patient level)		
Testing Data	**21**	Intended sample size	**Page 14** (Study Population)		

Section / Topic	No.	Item	Page / Line	No	NA
Model	**22**	Detailed description of model	**Pages 18-20** (Model Architecture)		
	**23**	Software libraries, frameworks, and packages	**Page 22** (Code Availability)		
	**24**	Initialization of model parameters	**Page 18** (Model Architecture)		
Training	**25**	Details of training approach	**Pages 20-21** (Training and Optimization)		
	**26**	Method of selecting the final model	**Page 21** (Early stopping...)		
	**27**	Ensembling techniques		**NA**	
Evaluation	**28**	Metrics of model performance	**Page 21** (Performance Evaluation)		
	**29**	Statistical measures of significance and uncertainty	**Page 21** (Performance Evaluation)		
	**30**	Robustness or sensitivity analysis	**Page 17** (Image Preprocessin g - Frame selection)		
	**31**	Methods for explainability or interpretability	**Page 20** (Model Architecture - Grad-CAM)		
	**32**	Evaluation on internal data	**Pages 5 & 21**		
	**33**	Testing on external data	**Pages 5 & 16**		
	**34**	Clinical trial registration		**NA**	
**RESULTS**					
Data	**35**	Numbers of patients or examinations included and excluded	**Pages 4-5** (Baseline Characteristic s)		

	**36**	Demographic and clinical characteristics of cases in each partition	**Page 5 & Page 27** (Table 1)		
Model performance	**37**	Performance metrics and measures of statistical uncertainty	**Pages 5-6 & Page 28** (Table 2)		
	**38**	Estimates of diagnostic performance and their precision	**Pages 5-6** (Clinical Evaluation of the Model)		
	**39**	Failure analysis of incorrect results	**Page 7** (Interpretability)		
**DISCUSSION**					
	**40**	Study limitations	**Pages 11-13** (Discussion)		
	**41**	Implications for practice, including intended use and/or clinical role	**Pages 9-10, 13** (Discussion)		
**OTHER INFORMATION**					
	**42**	Provide a reference to the full study protocol or to additional technical details	**Page 22** (Code Availability)		
	**43**	Statement about the availability of software, trained model, and/or data	**Pages 21-22** (Data/Code Availability)		
	**44**	Sources of funding and other support; role of funders	**Page 22** (Acknowledgments)		

* Indicate page and/or line number for each checklist item that is present. NA = not applicable.
